# The significant others of aurora kinase a in cancer: combination is the key

**DOI:** 10.1186/s40364-024-00651-4

**Published:** 2024-09-27

**Authors:** Kumar Nikhil, Kavita Shah

**Affiliations:** 1 Department of Chemistry, Purdue University Institute for Cancer Research, 560 Oval Drive, West Lafayette, IN 47907 USA; 2 School of Biotechnology, Kalinga Institute of Industrial Technology, Bhubaneswar, 751024 India

**Keywords:** AURKA, Cancer, Substrates, VHL, HURP, p53, ER, RASSF1, NFκB

## Abstract

AURKA is predominantly famous as an essential mitotic kinase. Recent findings have also established its critical role in a plethora of other biological processes including ciliogenesis, mitochondrial dynamics, neuronal outgrowth, DNA replication and cell cycle progression. AURKA overexpression in numerous cancers is strongly associated with poor prognosis and survival. Still no AURKA-targeted drug has been approved yet, partially because of the associated collateral toxicity and partly due to its limited efficacy as a single agent in a wide range of tumors. Mechanistically, AURKA overexpression allows it to phosphorylate numerous pathological substrates promoting highly aggressive oncogenic phenotypes. Our review examines the most recent advances in AURKA regulation and focuses on 33 such direct cancer-specific targets of AURKA and their associated oncogenic signaling cascades. One of the common themes that emerge is that AURKA is often involved in a feedback loop with its substrates, which could be the decisive factor causing its sustained upregulation and hyperactivation in cancer cells, an Achilles heel not exploited before. This dynamic interplay between AURKA and its substrates offers potential opportunities for targeted therapeutic interventions. By targeting these substrates, it may be possible to disrupt this feedback loop to effectively reverse AURKA levels, thereby providing a promising avenue for developing safer AURKA-targeted therapeutics. Additionally, exploring the synergistic effects of AURKA inhibition with its other oncogenic and/or tumor-suppressor targets could provide further opportunities for developing effective combination therapies against AURKA-driven cancers, thereby maximizing its potential as a critical drug target.

## Introduction

Aurora kinases belong to a family of serine/threonine kinases that act as key mitotic regulators for the even distribution of genetic material between daughter cells. Specifically, the Aurora kinases are critical for maintaining chromosome stability, duplication, maturation and separation of centrosomes, spindle assembly, and cytokinesis [1]. Aurora kinase was first identified in yeast, where it was christened as Ipl1 [2]. The first multicellular organism to report the Aurora kinase protein was Drosophila melanogaster, where it controlled monopolar spindle formation and centrosome separation [3]. Three years later, two Aurora kinase homologs were discovered in Caenorhabditis elegans [4, 5]. Since then, three different Aurora kinases have been discovered in mammals, namely Aurora A (AURKA), Aurora B (AURKB), and Aurora C (AURKC) [6]. While AURKA is predominantly located at the mitotic poles and controls bipolar spindle assembly during mitosis, Aurora B is primarily located at the spindle midzone and regulates cytokinesis and spindle-chromosome attachment [1]. AURKC is mainly expressed in the testis and controls spermatogenesis [7, 8].

Human Aurora kinases contain three domains: an N-terminal domain (39–139 amino acids), a protein kinase domain (250–300 amino acids), and a short C-terminal domain (15–20 amino acids) [7]. The N-terminal domain shares the least sequence homology and is mainly involved in protein-protein interactions. The kinase domain is highly conserved between Aurora proteins, with 71%, 60%, and 75% homology between AURKA/AURKB, AURKA/AURKC and Aurora B/C, respectively [8]. The C-terminal lobe of the kinase domain contains a conserved residue at T288 for AURKA, T232 for AURKB, and T195 for AURKC, whose phosphorylation induces a conformation change associated with the acquisition of kinase activity [9, 10]. The C-terminal domain of human Aurora B shares 53% and 73% sequence similarity with human AURKA and AURKC, respectively.

Overexpression of AURKA and AURKB occurs in cancers of many origins, where they function as oncogenes to promote tumorigenesis and metastasis. However, comparatively little information is available regarding the role of AURKC in cancer. Our review examines the most recent advances in AURKA regulation and its molecular mechanisms for promoting various cancers, with a particular focus on its disease-specific substrates and their associated signaling pathways.

## Mitotic and interphasic expression of AURKA

AURKA is predominantly recognized as a mitotic kinase, however, recent findings have uncovered an equally important role of interphasic AURKA in normal cells. In contrast, in cancer tissues, AURKA is overexpressed in a cell-cycle-independent manner and is mislocalized in the cytoplasm and/or nucleus depending upon the cancer.

Under physiological conditions, AURKA is differentially expressed during various phases of the cell cycle. It is present at relatively low levels during the G1 phase. However, during S-phase, it starts amassing at the centrosomes. In the late G2 phase, AURKA accumulates and gets activated at the centrosomes [11]. During metaphase, AURKA localizes at the spindle pole following nuclear envelope breakdown. The majority of AURKA is inactivated and degraded during late metaphase and early anaphase, however, a small fraction of AURKA remains on the centrosomes and the spindles at the onset of anaphase and localizes to the spindle midzone and centrosomes during late anaphase and telophase/cytokinesis [12, 13]. AURKA carries out divergent functions during mitosis such as centrosome maturation, mitosis entry, mitotic spindle formation, and cytokinesis [12, 13].

Pleiotropic roles of AURKA have also been discovered during the interphase, including its role in microtubule (MT) stabilization, neuronal outgrowth, ciliogenesis, and ATP production. During the G1 phase, AURKA localizes to the centrosomes and stabilizes MT following mitosis through Targeting protein for Xklp2 (TPX2) and centrosomal protein 192 (CEP192) [14]. AURKA also promotes MT stabilization via phospholipase D2 (PLD2) in interphase cells. PLD2 produces phosphatidic acid, which binds AURKA at the phosphatidic acid binding site (E171-E211), causing its activation and resulting in MT stabilization and cell migration [15]. In post-mitotic neurons, active AURKA promotes neuronal outgrowth by reorganizing the MT cytoskeleton [16]. Interestingly, using an AURKA-targeted PROTAC, a kinase-independent function of AURKA was revealed in promoting DNA replication in S phase [17].

Active AURKA is also present in proximity of cilia during the G0/G1 phase, where it favors the resorption of primary cilium, thereby triggering cell cycle entry [18]. The primary cilium, present in almost all types of vertebrate cells, is a microtubule-containing, hair-like organelle at the cell surface that senses and transduces various extracellular signals, and thus functions as osmosensor, mechanosensor and chemosensor. Assembly and disassembly of primary cilia are tightly coordinated throughout the cell cycle. While mitotic cells lack cilia, quiescent cells are ciliated. In most cells, resorption occurs in G1, when cells are preparing for active cycling, allowing cilia to act as checkpoints that prevent cells from entering cell cycle [19]. AURKA is a proximal component of the disassembly machinery during the G1 phase, where it interacts with several other proteins including HDAC6, promoting ciliary disassembly (described in detail in HDAC6 section). Impairment of these processes gives rise to many diseases, including polycystic kidney disease, Bardet-Biedl syndrome and cancer.

Interphasic AURKA is also imported and processed in mitochondria. Under physiological conditions, interphasic AURKA promotes mitochondrial fission, thereby maintaining optimal ATP levels for normal functions. In cancer, where AURKA is overexpressed, it leads to mitochondrial fusion, thus producing more energy to fuel rapidly proliferating cells [20].

## Regulation of AURKA activity

### Activation by allosteric activators and autophosphorylation

AURKA activity increases by 157-fold by autophosphorylation at T288 alone [21]. However, the cytoplasmic and microtubule phosphatases dephosphorylate it, rendering AURKA largely inactive. Nevertheless, during mitosis, AURKA is activated by several distinct mechanisms in response to different upstream signals, all of which culminate in its autophosphorylation at T288 in its T loop (aka activation loop). While autophosphorylation often leads to maximal activation for kinases, AURKA autophosphorylation only partially activates it. AURKA full activation requires binding to a variety of allosteric activators, including Ajuba, TPX2, Bora, and HEF1 to its smaller N-terminal kinase lobe. As AURKA is inhibited by binding its own N-terminal domain, activator binding releases this inhibition, activating AURKA. For most AGC family kinases, including protein kinase A (PKA) and RAC-alpha serine/threonine-protein kinase (AKT), the allosteric modulator is a linear peptide sequence within the kinase, but distal to the protein kinase domain. In contrast, for AURKA, the allosteric modulator is a separate protein. Equally importantly, these activators also uniquely recruit active AURKA at specific subcellular locations and protect it from phosphatases.

**Fig. 1:**
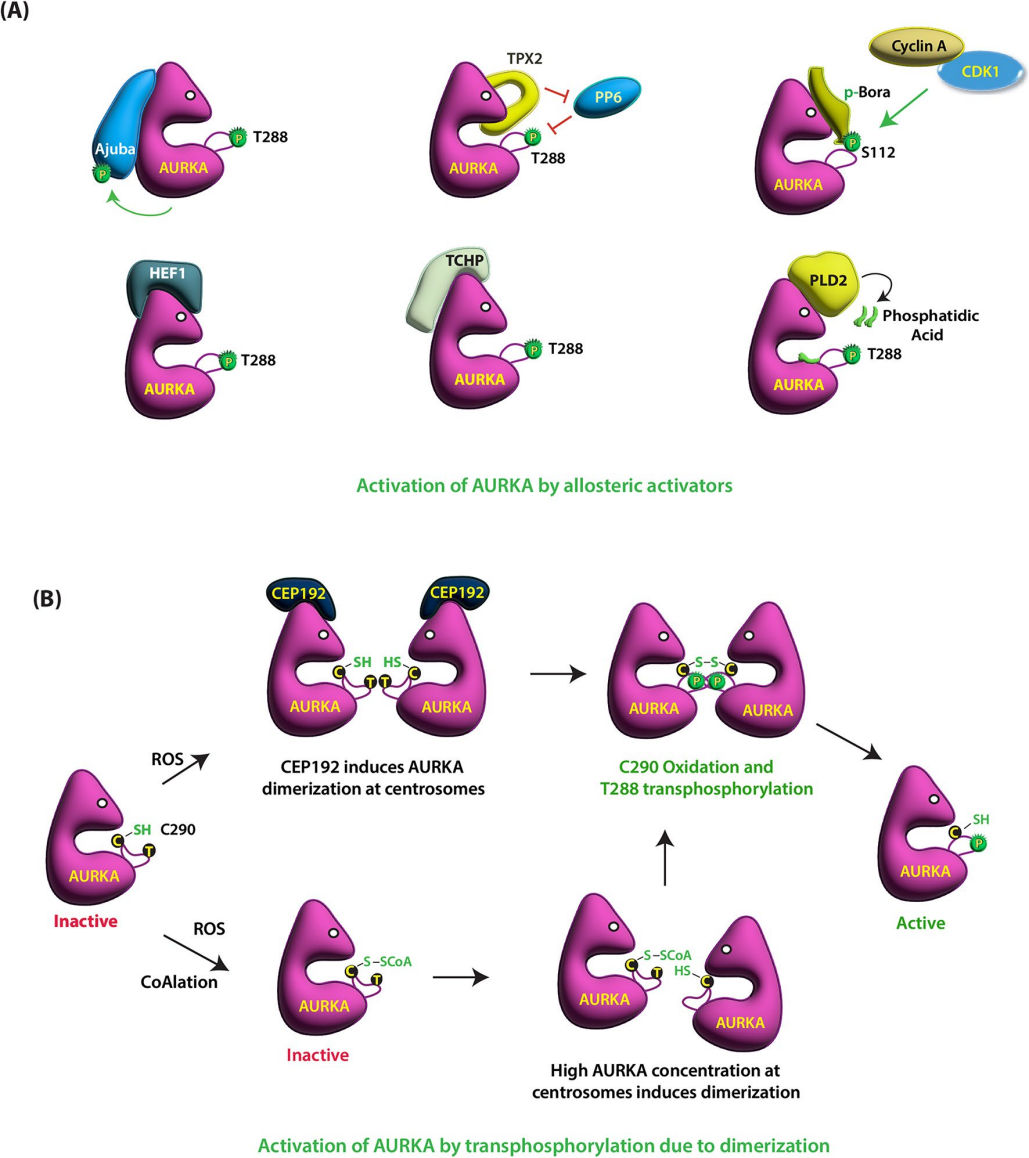
(**A**) Activation of AURKA by different allosteric activators. Ajuba binds to the N-terminal domain of AURKA causing its autophosphorylation at T288, which then phosphorylates Ajuba in return. TPX2 binding at the N-terminal domain of AURKA activates it and protects T288 dephosphorylation by PP6. TPX2 binding also protects it from CoAlation. CDK1/Cyclin A phosphorylates Bora at S112, which robustly activates unphosphorylated AURKA by binding it. HEF1 also binds and activates AURKA. TCHP binds and activates AURKA, although the exact mechanism of activation remains unclear. PLD2 binds AURKA, and generates phosphatidic acid, which binds AURKA at the phosphatidic acid binding site activating it. (**B**) Activation of AURKA in an organelle-specific manner. AURKA forms transient disulfide adduct formation at C290 in a CEP192-dependent manner under highly oxidative conditions, resulting in trans-autophosphorylation of T288 and robust activation at the centrosomes. AURKA also forms dimers at the centrosomes by oxidative modification of C290 by CoAlation. However, C290 oxidation of monomeric AURKA at other subcellular locations inactivates it due to low local concentration of AURKA

One of the first identified activators of AURKA was Ajuba, which has three tandem LIM domains at the C-terminus. Ajuba binds to the N-terminal segment of AURKA via its second and third LIM domains, resulting in AURKA autophosphorylation at T288, which then phosphorylates Ajuba in return (Fig. 1A) [11]. This event is essential for mitotic entry. Another activator of AURKA is the microtubule binding protein TPX2, which is released upon nuclear envelope fragmentation during mitosis. The N-terminal 1–43 amino acids of TPX2 binds to AURKA at two distinct sites, resulting in 15-fold activation. The first part (residues 7–21) binds at the N-terminal lobe of AURKA (Fig. 1A). The second part (30–43 residues) bind AURKA between N- and C-terminal lobes [22]. TPX2 binding also protects the dephosphorylation of T288 by PP6, resulting in the overall 448-fold activation of AURKA (Fig. 1A). TPX2 also recruits AURKA to the MTs, which then aids in spindle assembly. AURKA activation during mitosis also depends on Bora, following its own phosphorylation at S112 by CDK1/Cyclin A during the S/G2 transition [23]. Phosphorylated Bora binds and robustly activates unphosphorylated AURKA, suggesting that the S112 phosphate of Bora may compensate for the phosphorylated activation segment on AURKA (Fig. 1A).

AURKA is also activated during G1 by distinct activators including neural precursor cell expressed developmentally downregulated 9 (NEDD9 aka HEF1), centrosomal protein 55 (CEP55) and trichoplein (TCHP). Active AURKA promotes the disassembly of primary cilia during G1 phase, which is essential for cell cycle progression. The focal adhesion scaffolding protein HEF1 binds and activates AURKA during G1, which in turn phosphorylates HDAC6 causing cilia disassembly (details in HDAC6 section) [18]. CEP55, a centrosomal protein, does not directly activate AURKA, but binds and stabilizes it by facilitating its interaction with chaperonin CCT complex. AURKA stabilization results in disassembly of primary cilia in human RPE cells [24]. AURKA is activated by binding TCHP, a keratin intermediate filament scaffold protein, at the centrioles, although the exact mechanism of activation remains unclear. AURKA-binding to TCHP inhibits primary cilia assembly in G1 phase, thereby allowing cell cycle progression (Fig. 1A) [25]. AURKA directly binds PLD2, although the exact binding sites are not known. PLD2 produces phosphatidic acid, which activates AURKA via increased T288 phosphorylation [15] (Fig. 1A).

### Redox priming and dimerization— organelle-specific activation

In mammalian cells, overall ROS levels increase during the cell cycle, peaking in mitosis and resulting in the accumulation of oxidized protein cysteine residues [26]. During mitosis, AURKA molecules cluster across centrosomes that increases their local concentration at centrosomes. As AURKA possesses a conserved redox-sensitive C290 residue in its catalytic domain, the highly oxidative cytoplasm triggers transient disulfide adduct formation in a CEP192-dependent manner. The CEP192 promotes zinc-dependent face-to-face dimerization of centrosomal AURKA via disulfide bond formation at C290 residues during mitosis, resulting in trans-autophosphorylation of T288 and robust activation (Fig. 1B). Structural studies have shown that CEP192 binds at a different site in AURKA as compared to TPX2 [27]. AURKA also forms dimers at the centrosomes by oxidative modification of C290 by Coenzyme A (CoAlation). This in turn leads to its activation by autophosphorylation at T288 [28]. Interestingly, C290 CoAlation of monomeric AURKA at other subcellular inhibits its activity because its low local concentration at these sites does not support dimer formation (Fig. 1B) [29]. Redox priming thus ensures that AURKA activity is limited to specific cellular locations where dimer formation is favored. Importantly, TPX2 bound AURKA is protected from CoAlation, indicating that AURKA responds differentially to oxidation signals depending upon its subcellular locations, relative concentration, oligomerization status, and the presence of other binding partners.

## Expression and oncogenic role of AURKA

AURKA is an essential kinase, as the corresponding knock-out mice die before the 16-cell stage. In addition, germ line AURKA deficiency causes embryonic death at the blastocyst stage. AURKA also regulates meiotic maturation and fertilization of mouse oocytes [30]. AURKA is expressed in all somatic cells, particularly in dividing tissues including hematopoietic cells, colon, testis and mammary gland. AURKA is also expressed in non-dividing or slowly dividing tissues and exerts pleiotropic physiological functions during the interphase.

Amplification of the AURKA locus has been reported in a wide variety of tumors, including breast, prostate, pancreatic, ovarian, esophageal, colon, liver, lung, neuroblastoma, bladder and cervical cancer [31]. AURKA is also overexpressed in many cancers due to increased protein stability. For example, Inhibitor of differentiation 1 (ID1) overexpression inhibits AURKA degradation by competitively binding anaphase‑promoting complex/cyclosome Cdh1 (APC/CCdh1), which is responsible for AURKA ubiquitylation [32] AURKA can be overexpressed and constitutively activated due to upstream mitogenic signaling pathways like MAPK/ERK [33]. Activated MAPK signaling pathways induce AURKA accumulation in ERα^+^ breast cancer cells, leading to epithelial-to-mesenchymal transition (EMT) [33].

Overexpression of AURKA is strongly correlated with an increase in colony formation, centrosome amplification, tumorigenicity, EMT and stem cell formation [6, 12, 33]. However, overexpression of AURKA in primary cells is not a potent inducer of cellular transformation, suggesting that the oncogenic potential of AURKA derives from a sum of several spatially and temporally distinct actions.

Unlike normal cells, AURKA is localized beyond the nucleus in cancer cells, irrespective of cell cycle phase; where it can trigger a plethora of improper interactions, phosphorylation, and mislocalizations [34]. AURKA overexpression interferes with different cell cycle checkpoints, including the G1 checkpoint resulting in aneuploidy, the spindle assembly checkpoint preventing a defective spindle from reaching metaphase and anaphase, and the G2 checkpoint, which prevents cells with genetic aberration from entering mitosis [35]. AURKA overexpression also causes centrosome amplification and multinucleation resulting in abnormal mitotic spindles [36]. In addition, AURKA can interact with several tumor suppressor proteins (e.g. p53, BRCA1, Chfr) and inhibit their activities and/or expression, leading to tumor progression. AURKA overexpression also results in SMAD5 phosphorylation and its subsequent nuclear translocation, causing SOX2 upregulation and stem cell type phenotype acquisition [33]. However, it remains unclear whether SMAD5 is a direct or indirect substrate of AURKA. In this review, we examine 33 direct substrates of AURKA in various cancers, which have revealed the pleiotropic mechanisms by which they promote malignancy of different origins.

## Direct substrates of AURKA

### p53 and hnRNPK

**Fig. 2:**
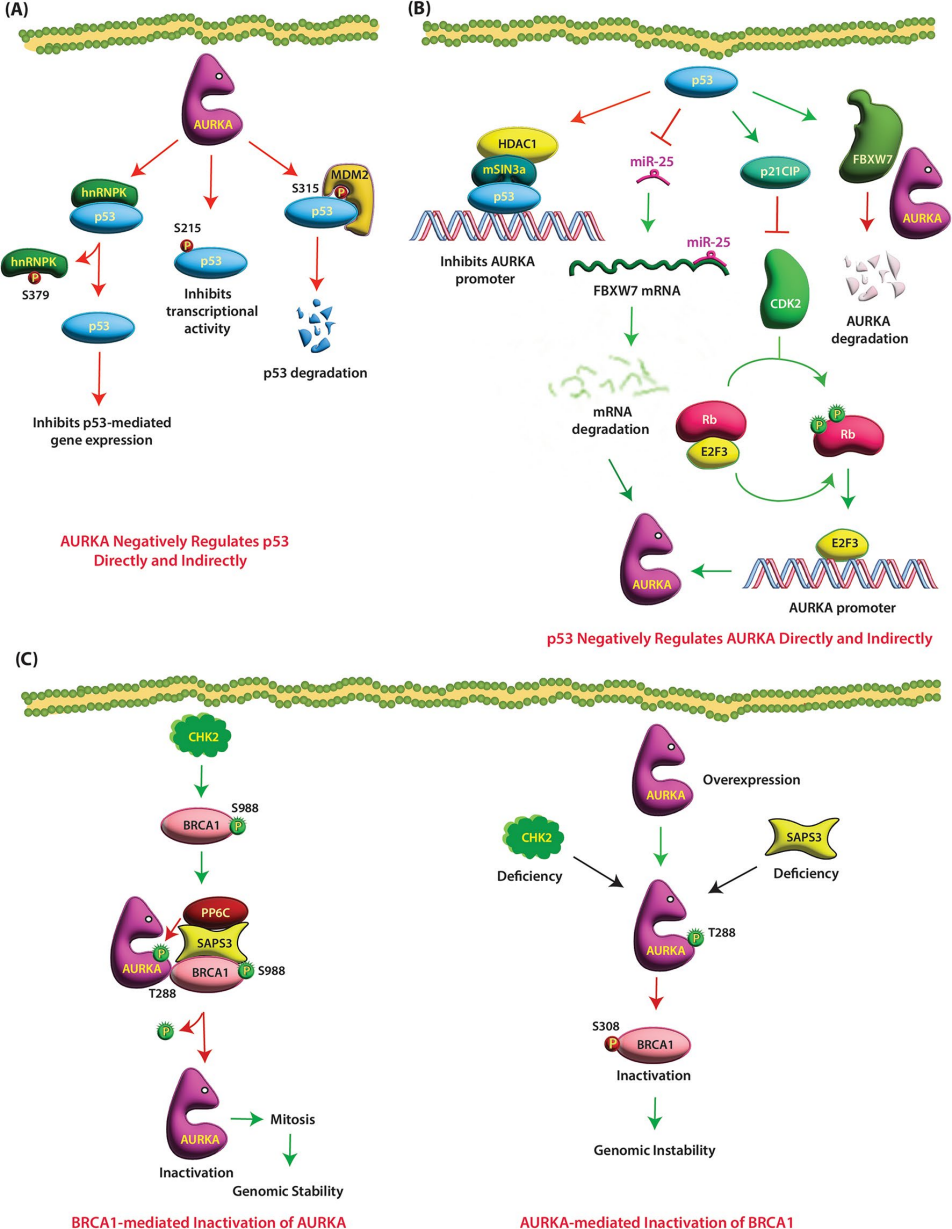
(**A**) AURKA negatively regulates p53. AURKA phosphorylates p53 at S215, which inhibits its transcriptional activity. AURKA also inhibits p53-mediated gene expression by phosphorylating its co-activator hnRNPK at S379, which disrupts its interaction with p53. AURKA triggers MDM2-mediated p53 degradation by phosphorylating p53 at S315. Red circles show inactivating phosphorylation events. (**B**) p53 negatively regulates AURKA transcription and protein stability. p53 in complex with mSIN3a and HDAC1 directly binds to AURKA promoter, thereby inhibiting AURKA transcription. p53 also inhibits AURKA transcription indirectly via CDK2. p53 increases p21CIP levels, which inhibits CDK2. In the absence of p53, CDK2 hyperphosphorylates Rb, which releases E2F3 increasing AURKA transcription. p53 facilitates AURKA degradation by increasing FBXW7 levels. p53 loss of function mutation increases the expression of miR-25, which downregulates FBXW7 expression, stabilizing AURKA protein. Green circles show activating phosphorylation events. (**C**) BRCA1 and AURKA inactivate each other. CHK2 phosphorylates BRCA1 at S988, which recruits the SAPS3-PP6C phosphatase complex, which dephosphorylates BRCA1-bound AURKA, thereby inhibiting its activity. AURKA overexpression or loss of CHK2 or PP6C results in AURKA-mediated BRCA1 phosphorylation at S308 which inactivates it. Green circles and arrows show activating phosphorylation and events, respectively

P53 is a transcription factor that inhibits tumor growth by regulating dozens of target genes possessing a myriad of biological functions. AURKA degrades p53 and inhibits its activity both directly and indirectly. AURKA phosphorylates p53 at S215 and inhibits its transcriptional activity (Fig. 2A) [37]. AURKA also phosphorylates p53 at S315, thereby allowing MDM2 to bind and degrade p53 [38] (Table 1). AURKA indirectly inhibits p53-mediated gene expression by phosphorylating heterogeneous nuclear ribonucleoprotein K (hnRNPK), a transcriptional coactivator of p53 on S379, which disrupts its interaction with p53 during DNA damage (Fig. 2A) (Table 1) [39].

**Table 1:** Direct downstream substrates of AURKA implicated in cancer

	Substrate	Physiological role	Phos. Site	Consequence of phosphorylation	Biological response	Reference
1	p53	Tumor Suppressor	S215 S315	Abrogates transactivation and enhance proteasomal degradation	Oncogenic transformation	[37], [38]
2	hnRNPK	Transcriptional coactivator of p53	S379	Inhibition of p53 by disrupting interaction between p53 and hnRNPK	Impairs p35-mediated gene transcription	[39]
3	BRCA1	Tumor Suppressor	S308	Increased mitotic MT assembly rate	Unstable karyotype	[43]
4	PHLDA1	Transcriptional activator	S98	PHLDA1 degradation	Increases oncogenic phenotypes in breast cancer cells	[48]
5	VHL	Tumor suppressor	S72	Inhibits VHL’s MT stabilizing activity	Aneuploidy and tumorigenesis	[51]
6	RASSF1A	Tumor Suppressor	T202 S203	Prevents mitotic arrest by degrading RASSF1A via APC/C-Cdc20	Cell cycle progression and tumorigenesis	[56], [57]
7	IκBα	Regulatory protein	S32	NFκB nuclear translocation and activation	Tumor growth and survival	[60]
8	TIFA	Adaptor protein	T9	Ubiquitination of TRAF6 and activation of IκK complex	Indirect activation of NFκB pathway	[62]
9	HURP	Mitotic spindle protein	S627 S725 S757 S830	Increases HURP stability	Indirect activation of NFκB pathway	[63]
10	FAF1	Tumor suppressor	S289 S291	Ubiquitin-independent, proteasome-dependent degradation of AURKA	Antagonizing AURKA-induced centrosome amplification and inducing G2/M arrest	[70]
11	ERα	Nuclear transcription factor	S167	Increased activation of ERα signaling	Tamoxifen resistance	[75]
12	GSK3β	Kinase	S9	Nuclear translocation of β-catenin and transcription of oncogenes	Increased gastric tumorigenesis and suppression of necroptosis	[79], [80]
13	β-Catenin	Transcriptional coregulator	S552 S675	Increased stability and transcriptional activity	Increased metastasis	[82]
14	HDAC6	Histone deacetylase	Unknown site	Increased deacetylation activity	Increased ciliary resorption and tumor progression	[18]
15	TWIST1	Transcription factor	S123 T148 S184	Increases its stability and transcriptional activity	EMT, chemoresistance and CSC phenotype	[85]
16	ALDH1A1	Enzyme	T267 T442 T493	Increases its oligomerization and dehydrogenase activity.	Increases protein stability, EMT and CSC phenotypes.	[88]
17	YBX1	Transcription and translational factor	T62 S102	Stabilizes YBX1 and promotes its nuclear translocation.	Increases EMT, chemoresistance and tumorigenesis	[94]
18	LIMK2	Kinase	S283 T494 T505	Inhibits LIMK2 degradation and increases its kinase activity.	Oncogenic phenotype and breast cancer malignancy	[98]
19	NKX3.1	Tumor suppressor	S28 S101 S209	Proteasomal degradation of SPOP	CRPC pathogenesis	[100]
20	SPOP	Adaptor protein	S33 T56 S105	ubiquitylation and proteasomal degradation of SPOP	Tumorigenesis, EMT and chemoresistance	[102]
21	SOX2	Transcription factor	S220 S251	Decreases the ratio of stem-cells during mitosis	Stem cell reprogramming	[103]
22	NuMa	Microtubule (MT)-binding protein	T1804 T1811 T1812 S1887 S1969 S2047 T2084 S2087	Increased cell survival	Prostate cancer cell proliferation	[104]
23	LDHB	Enzyme	S162	Reverses enzymatic activity, thereby promoting conversion of pyruvate to lactate	Tumorigenesis	[110]
24	RAL-A	GTPase	S194	Increased RALA activity and translocation from plasma membrane to mitochondrial membrane	Cell migration, anchorage-independent growth and mitochondrial fission	[111], [112]
25	KCTD12	Potassium channel protein	S200 S243	Activation of CDK1	Mitotic entry and tumorigenesis	[116]
26	LKB1	Kinase	S299	Impairs LKB1 interaction with AMPK.	Proliferation and migration	[117]
27	YAP	Transcriptional coregulator	S397	Increases its transcriptional activity, leading to aggressive oncogenic phenotypes	Proliferation and migration	[119]
28	NPM1	Multifunctional nucleolar protein	T95	Phosphorylated NPM1 directly binds YAP1, which increases YAP1 stability	Tumorigenesis	[121]
29	RPS6KB1	Kinase	T389	Increased catalytic activity of RPS6KB1	Increased proliferation and survival of KRAS-mutant gastrointestinal cancer cells	[123]
30	SDCBP	Adaptor protein	S131 T200	Stabilizes SDCBP and prevents EGFR internalization	Tumor growth	[124]
31	MMSET aka NSD2	Methyl transferase	S56	Stabilizes NSD2	Chemoresistance	[126]
32	SOX8	Transcription factor	S327	Increased nuclear localization	Promotes glucose metabolism and chemoresistance	[127]
33	KEAP1	Adaptor subunit of Cullin-3 E3 ubiquitin ligase	T80	Increased nuclear localization of Nrf2	Enhances tumorigenesis and ferroptosis resistance	[129]

Heterogeneous nuclear ribonucleoprotein K (hnRNPK); Pleckstrin homology-like domain, family A, member 1 (PHLDA1); Ras-associated domain family 1 isoform A (RASSF1A); Von Hippel Lindau (VHL); Inhibitors of NF-κB (IκBs); TRAF-interacting protein with the FHA domain (TIFA); FAS-associated Factor-1 (FAF1); Hepatoma upregulated protein (HURP); Glycogen synthase kinase (GSK)3β; Aldehyde dehydrogenase 1 (ALDH1A1); Y-box binding protein-1 (YBX1); LIM-domain kinase-2 (LIMK2); Nuclear mitotic apparatus (NuMa); Lactate dehydrogenase B (LDHB); Ral small GTPase (RalA); C-terminus of HSP70-interacting protein (CHIP); Potassium channel tetramerization domain containing 12 (KCTD12); Liver kinase B1 (LKB1); Yes-associated protein (YAP); Syndecan binding protein (SDCBP); Ribosomal protein S6 kinase B1 (RPS6KB1); Nucleophosmin1 (NPM1); Multiple Myeloma SET Domain Containing Protein (MMSET); Sex-determining region Y (SRY)-Box 8 (SOX8); Kelch-like ECH-associated protein 1 (KEAP1)

In a feedback loop, p53 inhibits AURKA both at the transcriptional and post-translational levels [40]. P53 indirectly inhibits AURKA transcription by impeding CDK2 activity via increased transcription of p21. Consequently, p53 depletion reduces p21 levels in cancer cells activating CDK2. Active CDK2 hyperphosphorylates retinoblastoma 1 (Rb1), causing its dissociation from E2F transcription factor 3 (E2F3). E2F3 subsequently binds to the AURKA gene promoter and enhances its expression (Fig. 2B) (Table 2) [40]. p53 also directly inhibits AURKA transcription by binding to its promoter in complex with histone deacetylase 1 (HDAC1) and mSin3a as corepressors (Fig. 2B) [41]. The p53-HDAC1-mSin3a repressive complex also inhibits AURKA expression upon cisplatin treatment [41]. Thus, the reciprocal relationship between AURKA and p53 has important implications for anticancer therapy.

**Table 2:** Upstream regulators of AURKA implicated in cancer

	Name	Regulation	Function and Mechanism	Reference
1	p53	Negative	p53 indirectly inhibits AURKA transcription by impeding CDK2 activity. p53 increases AURKA degradation by increasing E3 ubiquitin ligase FBXW7. p53 also directly inhibits AURKA transcription by binding to its promoter in complex with HDAC1 and mSin3a as corepressors. p53 loss of function mutation increases miR-25, which downregulates FBXW7 expression, increasing AURKA protein levels.	[40], [41], [42]
2	BRCA1	Negative	Normal mitotic MT assembly rate resulting in stable karyotype	[43]
3	PHLDA1	Negative	Induces AURKA degradation via ubiquitin-proteasomal degradation pathway and inhibits cell motility, proliferation and transformation in breast cancer cells.	[48]
4	VHL	Negative	VHL monoubiquitinates AURKA in a PHD-independent manner in quiescent cells, triggering AURKA degradation, thereby maintaining primary cilia.	[53]
5	FAF1	Negative	Ubiquitin-independent, proteasome-dependent degradation of AURKA.	[70]
6	ERα	Positive	Estrogen exposure of ERα-positive cells recruits transcription factor GATA-3, which binds to AURKA promoter increasing its transcription.	[77]
7	GSK3	Negative	Inhibits AURKA activity by mediating its phosphorylation at S349 site in Xenopus oocytes.	[81]
8	TWIST1	Positive	Stabilizes AURKA by preventing its degradation further leading to increased tumorigenesis and EMT in pancreatic cancer.	[85]
9	ALDH1A1	Positive	Upregulates AURKA by increasing its protein stability thereby triggering a reciprocal loop increasing aggressive oncogenic phenotypes in pancreatic cancer.	[88]
10	YBX1	Positive	Stabilizes AURKA, thereby promoting EMT, cancer stem cell phenotype and chemoresistance in CRPC.	[94]
11	LIMK2	Positive	Stabilizes AURKA, thereby promoting AURKA-mediated oncogenic pathways in breast cancer.	[98]
12	NKX3.1	Negative	NKX3.1 degrades AURKA in a reciprocal feedback loop.	[100]
13	SPOP	Negative	SPOP degrades AURKA via a feedback loop.	[102]
14	KCTD12	Positive	Increases AURKA activity by stimulating AURKA autophosphorylation at T288 via CDK1.	[116]
15	NSD2	Positive	NSD2 interacts with the N-terminal of AURKA and methylates it at K14 and K117, increasing its kinase activity and subsequent p53 degradation,	[125]

Pleckstrin homology-like domain, family A, member 1 (PHLDA1); Von Hippel Lindau (VHL); Glycogen synthase kinase (GSK)3β; Aldehyde dehydrogenase 1 (ALDH1A1); Y-box binding protein-1 (YBX1); LIM-domain kinase-2 (LIMK2); Potassium channel tetramerization domain containing 12 (KCTD12); Fas-associated Factor-1 (FAF1)

Furthermore, p53 increases AURKA degradation by increasing the expression of an E3 ubiquitin ligase named F-box and WD repeat-containing 7 (FBXW7) [42]. FBXW7 is considered as a critical tumor suppressor as it degrades many oncoproteins including AURKA. p53 loss of function mutation increases the expression of miR-25, which downregulates FBXW7 expression by binding its 3’-UTR, resulting in elevated levels of AURKA protein (Fig. 2B) (Table 2) [42].

### Breast cancer susceptibility proteins (BRCA)

BRCA1 is a multifunctional tumor suppressor protein that plays an important role in DNA damage response, DNA repair, chromatin regulation, and mitotic chromosome segregation. Loss of BRCA1 leads to chromosomal instability and aneuploidy. BRCA1 and AURKA associate and negatively regulate each other during mitosis. Mechanistically, BRCA1 is phosphorylated by CHK2 on S988 during mitosis, which ensures proper mitotic spindle assembly and chromosomal stability by inhibiting aberrant AURKA activity (Fig. 2C). BRCA1 phosphorylation at S988 by CHK2 recruits the SAPS3-PP6C phosphatase complex, which dephosphorylates BRCA1-bound AURKA, thereby inhibiting its activity. However, an increase in AURKA overexpression or loss of tumor suppressor CHK2 or PP6C leads to AURKA-mediated BRCA1 phosphorylation at S308 which inactivates it (Fig. 2C). Inactivation of BRCA1 causes increased rate of mitotic microtubule assembly, chromosome missegregation, and chromosomal instability (CIN) (Table 1) [43].

Another tumor suppressor of the same family, BRCA2 also plays an important role in maintaining genomic stability and inhibiting polyploidy. A negative correlation exists between AURKA and BRCA2 as well. AURKA is commonly overexpressed in breast cancers with *BRCA2* mutations [44]. Furthermore, overexpressed AURKA decreases BRCA2 expression in ovarian cancer [45]. However, the precise regulation mechanisms between AURKA and BRCA2 require further investigation.

### Pleckstrin homology-like domain, family A, member 1 (PHLDA1)

**Fig. 3:**
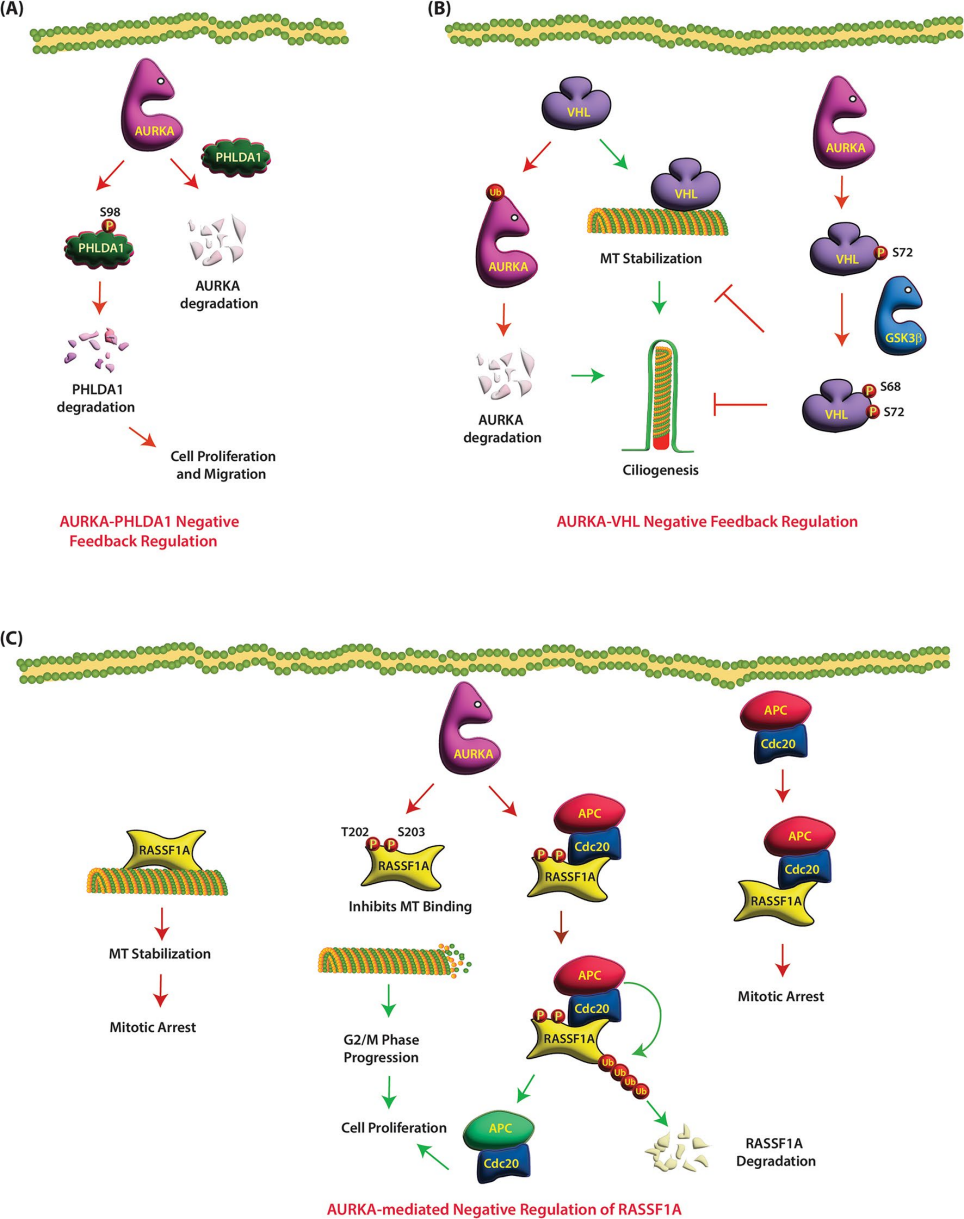
(**A**) AURKA and PHLDA1 negatively regulate each other. AURKA phosphorylates PHLDA1 at S98, leading to its degradation. PHLDA1 also negatively regulates AURKA levels, thereby triggering a feedback loop. Red circles and arrows show inactivating phosphorylation and events, respectively. (**B**) AURKA and VHL negatively regulate each other. VHL promotes ciliogenesis by stabilizing MTs. AURKA phosphorylates VHL at S72 position, which allows GSK3β to phosphorylate VHL at S68, which inhibits VHL’s MT stabilizing activity, thereby inhibiting ciliogenesis. VHL monoubiquitinates AURKA in quiescent cells, triggering AURKA degradation, thereby maintaining primary cilia. (**C**) AURKA negatively regulates RASSF1A. RASSF1A stabilizes MT causing mitotic arrest. Active AURKA phosphorylates RASSF1A at T202 and/or S203, allowing the cells to enter the M phase. RASSF1 also inhibits APC/C-Cdc20 activity by binding Cdc20 in early mitosis. AURKA-mediated phosphorylation of RASSF1 at T202 and S203, switches RASSF1 from an inhibitor of APC/C-Cdc20 complex to a substrate of APC/C-Cdc20. RASSF1 gets degraded, and the partially active APC/C-Cdc20 ubiquitylates Cyclin A, initiating the onset of anaphase

PHLDA1 is a multifunctional protein and a transcriptional activator that regulates a plethora of biological functions like apoptosis, cell proliferation, differentiation, and cell migration, depending on the cellular type and context [46]. PHLDA1 is expressed in various tissues with different subcellular localizations and functions, and its expression pattern is altered in different types of tumors [46]. PHLDA1 can act both as an anti-oncogene or an oncogene in a tissue-specific manner. Reduced PHLDA1 expression is seen in melanoma, breast carcinoma, oral carcinoma, and gastric adenocarcinoma, while upregulation was reported in colon cancer and in human intestinal adenoma and carcinoma [46, 47]. A regulatory loop between AURKA and PHLDA1 was reported in breast cancer cells by Johnson et al. (Fig. 3A) [48]. AURKA predominantly phosphorylates PHLDA1 at S98, leading to its degradation (Table 1). PHLDA1 also negatively regulates AURKA, thereby triggering a feedback loop (Table 2). Phosphorylation-resistant PHLDA1 strongly antagonizes AURKA-mediated oncogenic pathways, thereby revealing PHLDA1 degradation as a key mechanism by which AURKA promotes breast malignancy (Fig. 3A) [48].

### Von Hippel Lindau (VHL)

VHL is a tumor suppressor that plays pleiotropic roles in cells, including targeted protein degradation, ciliogenesis, extracellular matrix assembly, proliferation, survival, migration and cell polarity [49]. It serves as a substrate recognition module for an E3 ligase that targets various proteins, including HIF1α and HIF2α, for degradation. VHL recognizes most of its substrates including HIF1α and HIF2α upon hydroxylation at specific proline residues by prolyl hydroxylases (PHD) under normoxic conditions. However, under hypoxic conditions, PHD-triggered hydroxylation is inhibited, resulting in protein stabilization. VHL stabilizes MTs, including the MT axoneme of the primary cilium, thus playing a critical role in ciliogenesis. VHL loss results in a consistent loss of primary cilia, a disease categorized as ciliopathy.

VHL is commonly lost in the vast majority of clear cell renal cell carcinoma (ccRCC). ccRCC is the most common class of kidney cancer and accounts for 90–95% of all kidney cancers in adults. VHL loss is strongly associated with loss of primary cilia, renal cyst formation, and cancer. In contrast to VHL loss, AURKA levels are enhanced in ccRCC and in high-grade kidney tumors [50, 51]. Not surprisingly, AURKA and VHL share a bidirectional negative relationship. AURKA phosphorylates VHL at the S72 position in its β-domain, however, the exact consequence of this phosphorylation event was not analyzed in this study (Fig. 3B) (Table 1) [51]. Nevertheless, an earlier study reported that S72 phosphorylation is a prerequisite for GSK3β to phosphorylate VHL at S68, which in turn inhibits VHL’s MT stabilizing activity [52]. Thus, the authors speculated that defects in MT dynamics caused by AURKA-mediated phosphorylation of VHL could ultimately lead to aneuploidy and tumorigenesis by inhibiting ciliogenesis (Fig. 3B) [51].

In a reciprocal loop, VHL monoubiquitinates AURKA in a PHD-independent manner in quiescent cells, triggering AURKA degradation, which in turn is required to maintain the primary cilium (Fig. 3B) (Table 2) [53]. In contrast, tumor-associated variants of VHL are defective in ubiquitinating AURKA. AURKA inhibition thus rescues ciliogenesis in these cells. Subsequently, Dere et al. suggested that AURKA expression is driven by β-catenin-mediated transcription in VHL null cells [54]. VHL loss or depletion activates β-catenin and increases AURKA expression, which in turn decreases primary cilia formation and significantly shortens cilia length. Accordingly, VHL loss in RCC cells renders them highly sensitive to AURKA inhibitors [55].

### Ras-associated domain family 1 isoform A (RASSF1A)

RASSF1A is a microtubule- and centrosome-associated scaffolding protein with tumor suppressive functions. RASSF1A is frequently inactivated in human cancers due to its promoter methylation. Furthermore, Rassf1a (-/-) mice are vulnerable to developing both spontaneous and carcinogen-induced tumors. RASSF1A prevents cancer initiation, growth, and metastasis through many mechanisms including binding and stabilizing microtubules, resulting in cell cycle arrest in the M phase [56]. However, once AURKA is activated, it can alleviate this break by phosphorylating RASSF1A at T202 and/or S203, allowing the cells to enter the M phase [56], thereby restricting RASSF1A-mediated growth suppression in human tumors (Fig. 3C) (Table 1).

RASSF1 also regulates tumorigenesis by inhibiting anaphase-promoting complex (APC)-Cdc20 activity [57]. APC/cyclosome (APC/C) is a ubiquitin ligase that degrades mitotic proteins. APC/C is sequentially activated during the cell cycle by specific activators including Emi, Cdc20 and Cdh1. Emi activates APC/C during S phase and early mitosis (Fig. 3C). Cdc20 activates APC/C during mitosis and Cdh1 activates from anaphase to G1 phase. In early mitosis, RASSF1 inhibits APC/C-Cdc20 activity by binding Cdc20. However, AURKA-mediated phosphorylation of RASSF1 at T202 and S203 during mitotic progression, switches RASSF1 from an inhibitor of APC/C-Cdc20 complex to a substrate of APC/C-Cdc20 (Fig. 3C). Thus, RASSF1 gets degraded, and the partially active APC/C-Cdc20 ubiquitylates Cyclin A, initiating the onset of anaphase. AURKA-mediated phosphorylation of RASSF1 thus determines the time of degradation of APC/C substrates during mitosis (Table 1).

### IκBα: activation of the NF-κB pathway

AURKA influences the activities of numerous transcription factors for cancer progression. One such class of transcription factors called nuclear factor-kappa B (NF-κB) plays critical roles in proliferation, inflammation, immune response, and apoptosis. The NF-κB family has five members, namely NF-κB1 (p50), NF-κB2 (p52), RelA (p65), RelB, and c-Rel. These form homodimers and heterodimers, among which the heterodimer of p50 and p65 is the most common form [58]. Overactivation of the NF-κB pathway can lead to cancer, cardiovascular diseases, inflammatory diseases, and autoimmune diseases. Inhibitors of NF-κB (IκBs) include canonical IκB proteins (IκBα, IκBβ, and IκBε), non-canonical IκB proteins (Bcl-3, IκBζ, IκBNS, IκBη, and IκBL), and precursor IκB proteins (p105 and p100). IκB proteins bind NF-κB, which inhibit their activity by sequestering them in the cytoplasm.

**Fig. 4:**
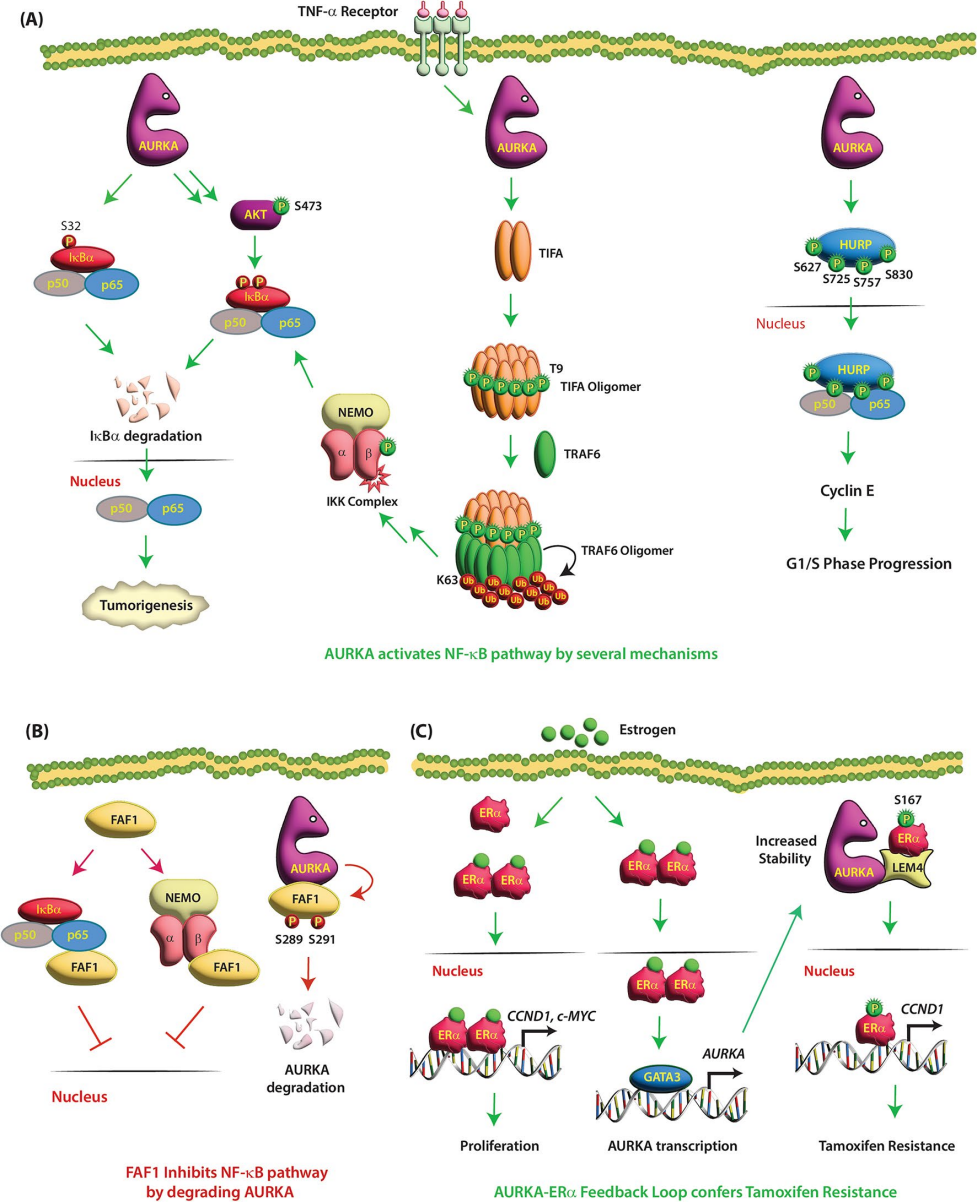
(**A**) AURKA activates NF-kB pathways by several mechanisms. AURKA phosphorylates IκBα at S32, leading to its degradation, thereby releasing NF-κB, which translocates to the nucleus, promoting tumorigenesis. AURKA also indirectly degrades IκBα via AKT. AURKA overexpression activates AKT, which degrades IκBα, thereby inducing NF-κB nuclear translocation to enhance cell survival. AURKA indirectly activates the NF-κB pathway by phosphorylating TIFA at T9, resulting in TIFA oligomerization. Phosphorylated TIFA interacts with TRAF6, ubiquitinating it. Ubiquitylated TRAF6 activates the IκK complex via TAK1 kinase, which also activates the NF-κB pathway. AURKA directly phosphorylates HURP at S627, S725, S757, and S830 increasing its stability. NF-κB also sequesters phosphorylated HURP in the nucleus, where HURP/NF-κB complex increases Cyclin E levels and facilitates G1 progression. (**B**) Phosphorylated FAF1 degrades AURKA, which inhibits NF-kB pathway. FAF1 directly binds NFκB and prevents its nuclear translocation. FAF1 also directly binds IKKβ, thereby inhibiting the NFκB pathway. AURKA phosphorylates FAF1 at S289 and S291, which allows phosphorylated FAF1 to degrade AURKA. (**C**) AURKA-ERα feedback loop confers tamoxifen resistance. Estrogen binding to ERα results in ERα dimerization and its recruitment to the promoters of ERα target genes, leading to proliferation. LEM4 binds both AURKA and ERα, which allows AURKA to phosphorylate ERα at S167, leading to tamoxifen resistance. Estrogen exposure of ERα-positive cells further recruits GATA-3, which binds to AURKA promoter increasing its expression in a feedback loop

Briassouli et al. revealed that AURKA mediates the phosphorylation of IκBα at S32 and S36, leading to its degradation. This releases NF-κB, which translocates to the nucleus, promoting tumor growth and survival (Fig. 4A) (Table 1) [59]. However, this study did not show whether AURKA directly phosphorylates IκBα. A subsequent report showed that AURKA directly binds IκBα and promotes its phosphorylation at S32 site, resulting in NF-kB activation [60]. Nonetheless, this study did not analyze whether AURKA also directly phosphorylates S36 site at IκBα.

AURKA also indirectly degrades IκBα via AKT, activating the NF-κB pathway [61]. In tongue squamous cell carcinoma (TSCC), a common type of head and neck squamous cell carcinoma, AURKA overexpression triggers AKT activation, which downregulates IκBα, subsequently inducing NF-κB nuclear translocation to enhance cell survival [61]. Accordingly, AURKA inhibition suppressed AKT activation, increased IκBα levels and down-regulated Bcl-xL expression, causing apoptosis (Fig. 4A).

### **TRAF-interacting protein with the FHA domain** (**TIFA): activation of the NF-κB pathway**

TIFA is the smallest known protein containing an FHA domain. The FHA domain binds phospho-threonine with high specificity. In acute myeloid leukemia (AML), AURKA indirectly activates the NF-κB pathway by phosphorylating TIFA at T9 (Table 1) [62]. Unphosphorylated TIFA exists as a dimer; however, upon TNFα stimulation, inter-molecular binding between phosphorylated T9 of TIFA dimers results in TIFA oligomerization. Phosphorylated TIFA interacts with TNF-receptor associated factor 6 (TRAF6), promoting its oligomerization, which activates TRAF6’s ubiquitin ligase activity causing self-ubiquitylation at K-63. Ubiquitylated TRAF6 activates the IκK complex via TAK1 kinase, which further activates the NF-κB pathway (Fig. 4A) [62].

### HURP

Hepatoma upregulated protein (HURP) is a cell cycle-regulated protein that is upregulated in many cancers. AURKA directly phosphorylates HURP at S627, S725, S757, and S830 increasing its stability and causing cell transformation (Table 1) [63]. Subsequent studies revealed that AURKA-mediated phosphorylation of HURP promotes its nuclear localization via NF-κB. NF-κB sequesters phosphorylated HURP in the nucleus, where HURP/NF-κB complex activates the cyclin E1 promoter. Thus, AURKA increases Cyclin E levels and G1 progression via the HURP/NF-κB complex (Fig. 4A) [64].

### FAS-associated Factor-1 (FAF1): an inhibitor of NFκB pathway

FAF1, initially identified as a member of the FAS death-inducing signaling complex, is an evolutionary conserved tumor suppressor that promotes apoptosis through several mechanisms. FAF1 contains several protein-interaction domains, including a death effector domain-interacting domain (DEDID), a FAS-interacting domain (FID), and a multi-ubiquitin-related domain at the C-terminus [65]. FAF1 is a negative regulator of NFκB pathway. FAF1 directly binds NFκB and prevents its nuclear translocation upon stimulation by tumor necrosis factor (TNF)-α, interleukin-1β, or lipopolysaccharide [66]. Similarly, FAF1 also directly binds IKKβ, thereby disrupting the IKK complex and inhibiting the NFκB pathway (Fig. 4B) [67]. FAF1 enhances caspase-8 assembly in response to the Fas signal [68] and promotes chemotherapeutic-induced apoptosis by death effector filament formation [69].

AURKA phosphorylates FAF1 at S289 and S291, which allows phosphorylated FAF1 to trigger ubiquitin-independent, proteasome-dependent degradation of AURKA, thereby antagonizing AURKA-induced centrosome amplification and inducing G_2_/M arrest (Tables 1 and 2) [70]. Interestingly, AURKA-mediated phosphorylation of FAF1 does not impact FAF1 levels. As a result, phospho-mimetic FAF1-DD mutant sensitizes cells to taxol, presumably by reducing AURKA levels. As AURKA promotes NFκB pathway by several mechanisms, FAF1-mediated AURKA degradation also inhibits NF-κB activation (Fig. 4B).

### **Estrogen receptor α (ERα)**

ERα is a hormone-dependent nuclear transcription factor that plays an important role in the development and progression of breast cancer [71]. Estrogen binding to ERα results in ERα dimerization and its recruitment to the estrogen-responsive elements (EREs) on the promoters of ERα target genes, leading to cell proliferation (Fig. 4C) [71]. Targeting ERα using the selective estrogen receptor modulator tamoxifen is the most widely used endocrine therapy, which inhibits breast cancer growth through competitive binding of ERα [71]. AURKA promotes distant metastases in ERα-positive breast cancer cells [33, 72] and its overexpression is strongly linked to decreased survival, high nuclear grade, and elevated HER-2/neu and progesterone receptor levels in breast cancer [73, 74].

AURKA phosphorylates and activates ERα via LEM4 in ER^+^ breast cancer cells (Fig. 4C) [75]. LEM proteins are nuclear envelope proteins that interact with barrier-to-autointegration factor (BAF) and connect interphase chromosomes to the nuclear lamina, thereby contributing to global nuclear organization [76]. LEM4 upregulation accelerates malignant cell growth in breast tumorigenesis and alters the cell cycle by promoting the G1/S phase transition [75]. LEM4 overexpression in ER^+^ breast cancer cells confer estrogen independence and tamoxifen resistance through activation of both the cyclin D-CDK4/6- Retinoblastoma-1 (Rb) pathway and ERα signaling [75]. LEM4 acts as a scaffold for both AURKA and ERα, which allows AURKA to phosphorylate ERα at S167, leading to the activation of ERα signaling (Fig. 4C) (Table 1) [75]. Furthermore, LEM4 stabilizes both ERα and AURKA further potentiating tamoxifen resistance. AURKA is also transcriptionally upregulated by ERα (Table 2) [77]. Estrogen exposure of ERα-positive cells recruits transcription factor GATA-3, which binds to AURKA promoter increasing its expression. Thus, AURKA-ERα feedback signaling confer tamoxifen resistance (Fig. 4C). As a result, AURKA is being actively pursued as a therapeutic target in breast cancer endocrine therapy.

### GSK3β

**Fig. 5:**
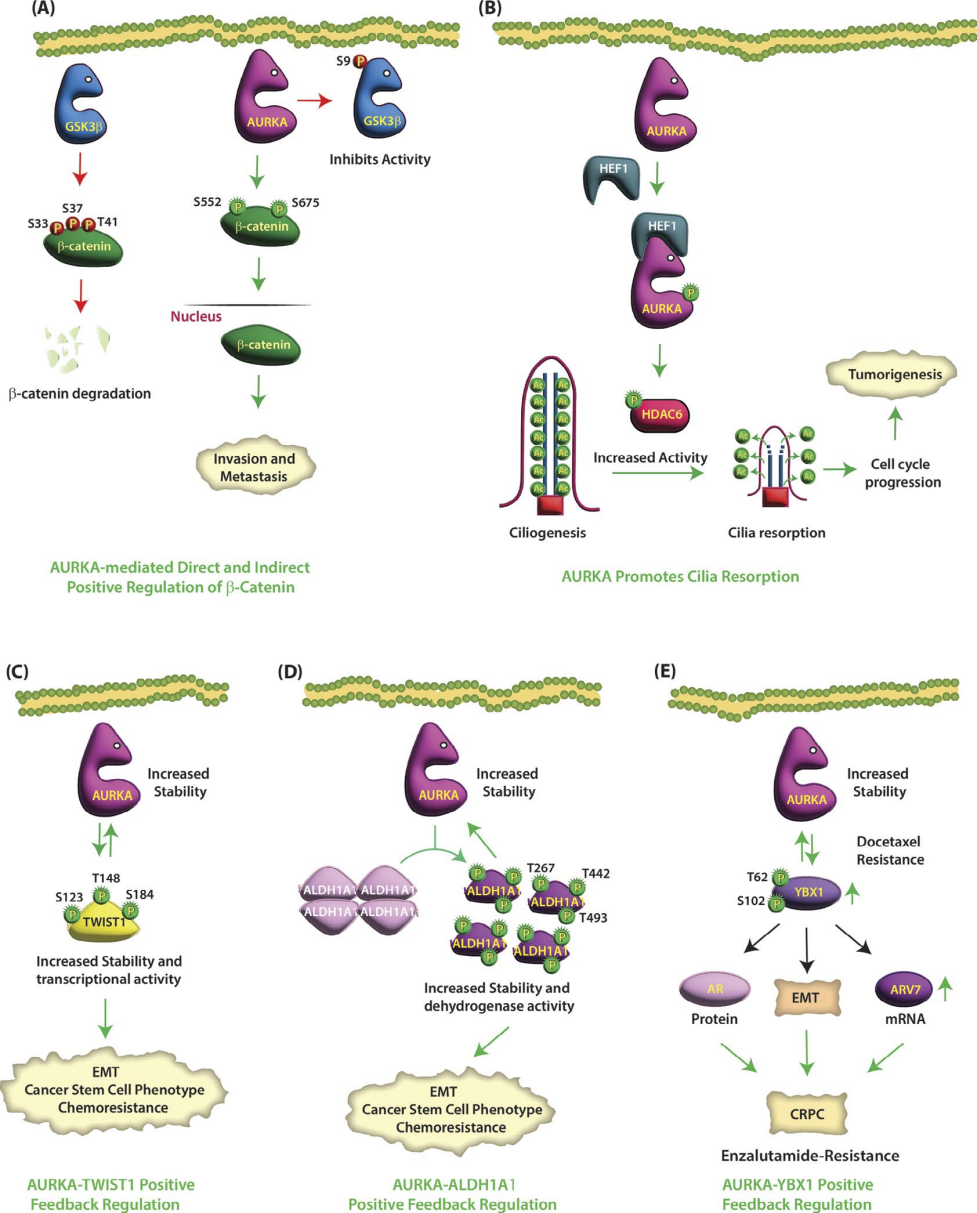
(**A**) AURKA positively regulates β-Catenin levels directly by phosphorylation and indirectly via GSK3β. GSK3β phosphorylates β-catenin at S33, S37, and T41, causing its degradation, thereby inhibiting cancer. AURKA phosphorylates GSK3β at S9, which inhibits its activity, leading to the nuclear translocation of β-catenin. AURKA directly phosphorylates β-catenin at the S552 and S675 sites, which increases its stability, enhances its cytoplasmic and nuclear expression, and increases its transcriptional activity, leading to metastasis. (**B**) AURKA promotes cilia resorption via HDAC6. HEF1 binding to AURKA changes its conformation, allowing it to undergo autophosphorylation and activation. Thus, growth factor-dependent upregulation of HEF1 activates AURKA, which in turn phosphorylates HDAC6 increasing its deacetylation activity. HDAC6-mediated deacetylation of MTs induces ciliary resorption, thereby promoting tumor progression. (**C**) AURKA-TWIST1 axis promotes EMT and chemoresistance in CRPC. AURKA phosphorylates TWIST1 at S123, T148, and S184, which increases its stability and transcriptional activity, resulting in tumorigenesis. TWIST1 also stabilizes AURKA in a feedback loop. (**D**) ALDH1A1-AURKA feedback loop promotes EMT, CSC and tumorigenesis. AURKA phosphorylates ALDH1A1 at T267, T442, and T493, which dissociates tetrameric ALDH1A1 into a highly active monomeric species. AURKA-dependent phosphorylation of ALDH1A1 also increases its enzymatic activity and protein levels. ALDH1A also upregulates AURKA by increasing its protein stability thereby triggering a reciprocal loop. (**E**) AURKA-YBX1 feedback loop promotes EMT, CSC and tumorigenesis. AURKA phosphorylates YBX1 at T62 and S102, which increases its protein stability. YBX1 reciprocates and stabilizes AURKA, thereby initiating a synergistic crosstalk

The Glycogen synthase kinase (GSK)3β is a cytosolic kinase that restricts β-catenin levels in cells. β-Catenin is a multifunctional factor that upregulates numerous genes critical for malignancy. Increased accumulation of β-catenin in the cytoplasm triggers its nuclear translocation, where it binds with the TCF/LEF family of transcription factors, increasing the transcription of oncogenic genes. GSK3β phosphorylates β-catenin at S33, S37, and T41, causing its ubiquitination and proteasomal degradation, thereby inhibiting cancer (Fig. 5A) [78]. AURKA and GSK3β interact with each other in gastric cancer cells, thereby enabling AURKA to phosphorylate GSK3β at S9, which inhibits its activity (Fig. 5A) (Table 1) [79]. This interaction thus leads to the nuclear translocation of β-catenin and the activation of oncogenic genes. Xie et al. further showed that AURKA inhibition induces necroptosis in pancreatic carcinoma via GSK3β [80]. Necroptosis is a caspase-independent form of regulated cell death that is induced by specific stimuli in apoptosis-resistant cells. Genetic or pharmacological inhibition of AURKA significantly inhibited GSK3β S9 phosphorylation in pancreatic cancer cells, suggesting that GSK3β might indeed act as a downstream target of AURKA in necroptosis.

Interestingly, in Xenopus oocytes, GSK3 has been shown to be a negative regulator of AURKA. GSK3 phosphorylates AURKA at the S290 and S291 sites, which primes the phosphorylation of AURKA at the S349 site (Table 2). As the S349 site is not a GSK3 consensus phosphorylation site, the authors speculated that it may be an autophosphorylation site for AURKA, although it was not conclusively demonstrated [81]. Nevertheless, S349 phosphorylation indeed inhibited AURKA kinase activity, since the (S349A)AURKA mutant was constitutively active despite GSK3-mediated phosphorylation at the S290 and S291 sites. Whether such a mechanism exists in human cells needs further investigation.

### β-Catenin

In addition to GSK3β-mediated β-catenin regulation, AURKA also directly binds β-catenin in esophageal squamous cell carcinoma (ESCC) cells. Normal tissues express weak or negative expression of AURKA, but in the vast majority of ESCC (73%) tissues, increased levels of cytoplasmic AURKA are reported [82]. AURKA overexpression is strongly linked with differentiation grades, metastasis, and distant lymph node metastasis in ESCC. In normal tissues, similar to AURKA, β-catenin levels are also low and localized to the plasma membrane. However, in ESCC tissues, increased cytoplasmic β-catenin expression was observed in 73% of cases (out of 129). More importantly, higher AURKA expression is positively correlated with increased cytoplasmic β-catenin expression, indicating AURKA overexpression causes the accumulation of cytoplasmic β-catenin in ESCC. The authors indeed confirmed that AURKA overexpression leads to increased cytoplasmic and nuclear expression of β-catenin. Mechanistically, AURKA directly phosphorylates β-catenin at the S552 and S675 sites, which increases its stability, enhances its cytoplasmic and nuclear expression, and increases its transcriptional activity, leading to metastatic potential in vitro and in vivo (Table 1) (Fig. 5A).

### HDAC6— inhibition of ciliogenesis

Histone deacetylase 6 (HDAC6) overexpression in cancer promotes cell migration and invasion by proteolytic degradation of the extracellular matrix. Similarly, human enhancer of filamentation 1 (HEF1/Cas-L/Nedd9) is a scaffolding protein that is overexpressed in multiple tumor types and is an established marker of malignancy. HEF1 both activates and stabilizes AURKA. HEF1 binds and changes the conformation of AURKA, allowing it to undergo autophosphorylation and activation (Figs. 1 and 5B). HEF1 stabilizes AURKA by inhibiting its binding to APC/C ubiquitin ligase [83]. Thus, HEF1 overexpression phenocopies AURKA overexpression and leads to increased centrosomes and multipolar spindles, resulting in aneuploidy [83]. In quiescent cells, AURKA and HEF1 are present at low levels and localize at the basal body, allowing MT assembly and cilia growth, thereby preventing cell cycle entry. However, growth factor-dependent upregulation of HEF1 activates AURKA, which in turn phosphorylates HDAC6 at an unknown site, which increases its deacetylation activity (Table 1). HDAC6-mediated deacetylation of MTs induces ciliary resorption, thereby promoting cell cycle entry and subsequent tumor progression (Fig. 5B) [18].

### TWIST1

TWIST1 is a highly conserved basic helix-loop-helix (bHLH) transcription factor [84]. TWIST1 expression is essential for embryonic development; however, it is downregulated after birth and is restricted to quiescent adult stem cells located in mesoderm-derived mesenchymal tissues. TWIST1 upregulation occurs in a variety of human cancers. TWIST1 promotes tumorigenesis by inhibiting differentiation, interfering with the p53 pathway, favoring cell survival, and/or inducing EMT [84]. Wang et al. identified a crucial role of the AURKA-TWIST1 axis in promoting EMT and chemoresistance in pancreatic cancer [85]. TWIST1 was identified as the first EMT-specific target of AURKA. AURKA phosphorylates TWIST1 at three different sites (S123, T148, and S184), which increases its stability and transcriptional activity, resulting in enhancement of cell motility, drug resistance, and the acquisition of a stem cell-like phenotype (Fig. 5C) (Table 1) [85]. Interestingly, these post-translational modifications also created a feedback loop as the levels of AURKA rose upon TWIST1 overexpression (Table 2) [85]. However, future studies are needed to explore whether AURKA-TWIST1 signaling plays a role in EMT paradigm beyond pancreatic cancer.

### ALDH1A1

ALDH1A1 is a cytosolic enzyme that catalyzes the oxidation of various aldehydes, including *trans*- and *cis*-retinal, to their corresponding less reactive acids, thereby detoxifying these compounds [86]. ALDH1A1 also binds to endobiotics and xenobiotics and possesses antioxidant activity. It is highly expressed in normal as well as cancer stem cells (CSCc) and thus is widely used as a marker to identify and isolate various types of CSCs [87]. While several studies have shown increased mRNA or protein levels of ALDH1A1 in different cancers, the molecular mechanism remains unclear. Wang et al. showed that AURKA and ALDH1A1 associate in pancreatic cancer cells and co-localize in pancreatic tissues. AURKA phosphorylates ALDH1A1 at three critical residues (T267, T442, and T493) and exerts multifaceted regulation over its subcellular localization, levels, enzymatic activity, and quaternary structure (Fig. 5D) (Table 1) [88]. AURKA-mediated phosphorylation rapidly dissociates tetrameric ALDH1A1 into a highly active monomeric species. AURKA-dependent phosphorylation of ALDH1A1 also increases its stability, thereby causing a dramatic enhancement in cell motility, chemoresistance, CSC phenotype, and EMT (Fig. 5D) [88]. In return, ALDH1A also upregulates AURKA by increasing its protein stability thereby triggering a reciprocal loop increasing aggressive oncogenic phenotypes (Table 2).

### Y-box binding protein 1 (YBX1)

YBX1 is a multifunctional oncoprotein that functions as a transcriptional and translational factor to regulate its target gene expression [89]. YBX1 is overexpressed in a wide variety of human cancers, such as prostate, colon, breast, lung, gastric, esophageal, and glioblastoma [89, 90]. YBX1 is known to deregulate a wide range of genes and proteins involved in cell proliferation, DNA replication, DNA repair, multi-drug resistance, and EMT [90]. YBX1 is phosphorylated by different kinases, leading to its activation and tumorigenesis [91–93]. AURKA phosphorylates YBX1 at two key residues (T62 and S102), which results in its nuclear translocation and increased protein stability, leading to aggressive malignant phenotypes including EMT, CSC and chemoresistance in castration resistant prostate cancer (CRPC) cells (Fig. 5E) (Table 1) [94]. YBX1 reciprocates and stabilizes AURKA by hindering its ubiquitylation, thereby initiating synergistic crosstalk (Fig. 5E) (Table 2).

### LIM kinase 2 (LIMK2)

**Fig. 6:**
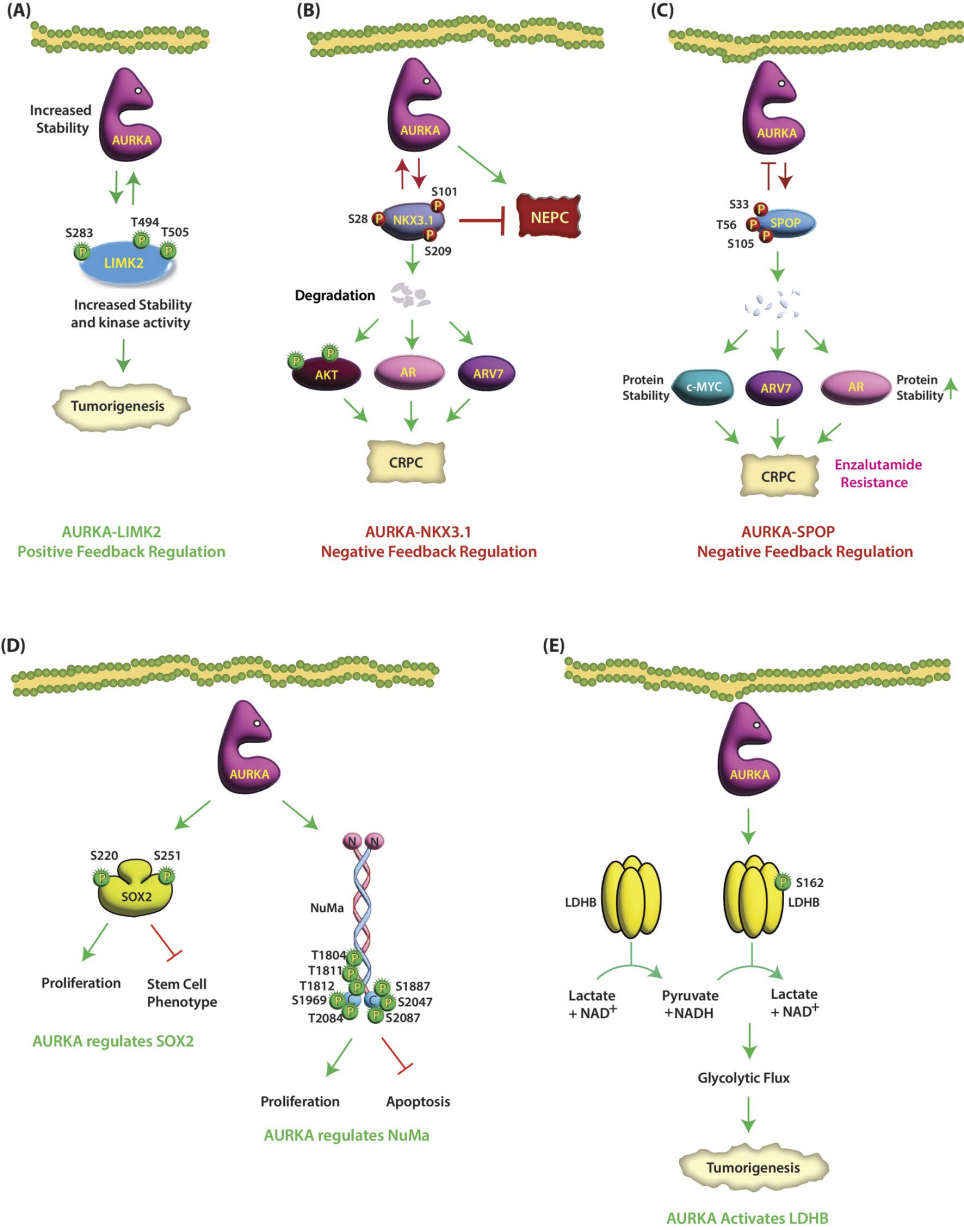
(**A**) AURKA and LIMK2 are involved in positive feedback loop. AURKA directly phosphorylates LIMK2 at S283, T494, and T505 in cancer cells. Similarly, LIMK2 also positively regulates the levels of AURKA. (**B**) AURKA and NKX3.1 negatively regulate each other. AURKA phosphorylates NKX3.1 at S28, S101 and S209, which ubiquitylates it. NKX3.1 also degrades AURKA in a reciprocal feedback loop. (**C**) AURKA and SPOP negatively regulate each other. AURKA directly phosphorylates SPOP at S33, T56 and S105, triggering its ubiquitylation. SPOP also degrades AURKA via a feedback loop. (**D**) AURKA regulates SOX2-based signaling. AURKA directly phosphorylates SOX2 at S220 and S251, which promotes proliferation, but inhibits stem cell phenotype. (**E**) AURKA promotes proliferation via NuMa. AURKA phosphorylates NuMa at multiple positions promoting cell proliferation and inhibiting apoptosis. (**F**) AURKA reverses the activity of LDHB via phosphorylation. AURKA phosphorylates LDHB at S162 and switches its enzymatic activity in the opposite direction

LIMK2, a member of the LIM kinase family, is a serine/threonine, and sometimes a tyrosine kinase. It is highly expressed in the brain, kidney, lung, stomach, and testis [95]. Since LIMK2 is associated with cytoskeleton dynamics and motility mechanisms, it plays an essential role in tumorigenesis, tumor-cell invasion, and metastasis [96]. LIMK2 phosphorylation at T505 by Rho-associated protein kinase (ROCK) is essential for initial LIMK2 activation [97]. Johnson et al. showed that LIMK2 is a critical substrate of AURKA, where AURKA regulates LIMK2 kinase activity, subcellular localization, and protein levels by direct phosphorylation at S283, T494, and T505 in breast cancer cells (Fig. 6A) (Table 1) [98]. Similarly, LIMK2 also positively regulates the levels of AURKA, thereby engaging in a positive feedback loop and promoting AURKA-mediated oncogenic pathways (Fig. 6A) (Table 2). Since AURKA is overexpressed in various types of cancer, analysis of LIMK2 and AURKA levels could supplement standard staging information in primary biopsy samples. LIMK2 inhibition or ablation can be considered as an alternative approach to modulating AURKA deregulation in cancer.

### NKX3.1

NKX3.1 is a prostate-specific tumor suppressor, which is either lost or its protein levels are drastically downregulated in prostate cancer (PCa) tissues. NKX3.1 downregulation is invariably associated with poor prognosis in PCa [99]. AURKA phosphorylates NKX3.1 at S28, S101 and S209, which degrades it, however, AURKA does not regulate NKX3.1 mRNA level (Table 1) [100]. NKX3.1 ubiquitylation promotes highly aggressive oncogenic phenotypes in cells. NKX3.1 also degrades AURKA in a reciprocal feedback loop. NKX3.1-AURKA loop in turn upregulates AR, AKT and ARv7 signaling driving highly malignant phenotypes (Fig. 6B) (Table 2). NKX3.1 overexpression also inhibits neuroendocrine phenotypes in neuroendocrine PCa (NEPC) cells. Thus, loss of NKX3.1 could be a major mechanism leading to AURKA upregulation in CRPC and NEPC and vice versa (Fig. 6B) [100].

### Speckle-type POZ (pox virus and zinc finger) protein (SPOP)

SPOP is an adaptor protein for E3 ubiquitin ligase, which can serve as an oncogene or an anti-oncogene. SPOP is the most mutated gene in CRPC (15%), which strongly associates with poor prognosis [101]. Furthermore, 85% of remaining tumors possessing wild-type SPOP still show reduced protein levels, underscoring that SPOP downregulation is an essential step in CRPC progression. AURKA directly phosphorylates SPOP at S33, T56 and S105, triggering its ubiquitylation (Table 1) [102]. SPOP degradation stabilizes AR, ARv7 and c-Myc, driving aggressive oncogenic phenotypes. SPOP also degrades AURKA via a feedback loop (Table 2). Accordingly, phospho-resistant SPOP fully inhibits tumorigenesis and EMT in vivo and renders cells sensitive to enzalutamide (Fig. 6C) [102].

### SOX2

The transcription factor SOX2 is critical for stem cell pluripotency and self-renewal and is essential during embryogenesis. SOX2 expression also occurs in adult stem cells (ASCs) but is downregulated in all other adult cells. However, SOX2 is overexpressed in most cancers and is highly detrimental. SOX2 overexpression is strongly associated with CSC, EMT, poor prognosis, and chemoresistance in numerous human cancers. AURKA directly phosphorylates SOX2 at S220 and S251, which decreases the ratio of stem cells during mitosis (Fig. 6D) (Table 1) [103]. As both AURKA and SOX2 are overexpressed in numerous cancers, the impact of their potential crosstalk on aggressive oncogenic phenotypes remains a subject of huge interest.

### Nuclear mitotic apparatus (NuMa)

NuMa was identified as a substrate of AURKA by Toughiri et al. [104]. NuMa is a ubiquitously expressed protein that is a component of the interphase nucleus and the mitotic spindle pole matrix [105]. NuMa promotes spindle assembly by associating with microtubule motors [106]. It also aids in spindle positioning during asymmetric cell division [107]. NuMa is overexpressed in many cancers and is associated with mitotic defects, poor prognosis and aneuploidy [108, 109]. As aneuploidy is a common feature of tumors that drives tumor progression, the role of NuMa in cancer has recently become a subject of renewed interest. AURKA phosphorylates the C-terminal of NuMa at multiple positions, such as T1804, T1811, T1812, S1887, S1969, S2047, T2084, and S2087 (Fig. 6D) (Table 1). Functional analyses demonstrated that phospho-resistant mutations of some of these sites significantly diminished cell proliferation and significantly increased the rate of apoptosis, leading the authors to hypothesize that NuMa may promote cancer via AURKA-mediated phosphorylation, although a direct role of AURKA in NuMa-mediated cancer was not investigated in this study [104].

### Lactate dehydrogenase B (LDHB)

Tetrameric LDH includes LDHA and LDHB, two subunits that are encoded by independent genes. LDHA catalyzes the conversion of pyruvate to lactate and regenerates NAD^+^, whereas LDHB catalyzes the opposite reaction and converts lactate to pyruvate along with the formation of NADH. AURKA binds LDHB and phosphorylates it at S162 and switches its enzymatic activity in the opposite direction (Table 1). Phosphorylated LDHB instead converts pyruvate to lactate, which efficiently promotes NAD^+^ regeneration, glycolytic flux, lactate production, and biosynthesis with glycolytic intermediates, which facilitate tumor progression [110]. Blocking LDHB phosphorylation by the expression of the (S162A) LDHB mutant inhibits glycolysis and tumor growth in cancer cells and xenograft models (Fig. 6E). These results reveal a pathway by which AURKA promotes the Warburg effect via post-translational modification of LDHB [110].

### RAL-A

The RAS-like oncoproteins A and B (RAL-A and RAL-B) are small G proteins that are key effectors of RAS. RAS directly binds and activates RAL guanine nucleotide exchange factors (RAL-GEFs), which in turn switch-on RAL-A/B activities by promoting GTP loading. RAL-A and RAL-B regulate a plethora of physiological functions, including mitochondrial fission, filopodia formation, vesicle trafficking, and cytokinesis. RALs are also critical mediators of cancer cell survival, invasion, migration, and metastasis. Although RAL-A and RAL-B share 80% sequence identity and nearly 100% identity in their effector domains, they often display striking functional disparities in cancer. While RAL-A is important for tumor initiation and growth, RAL-B promotes survival and metastasis. RAL-A and RAL-B are mainly different at their C-terminus, a 30-residue hypervariable region that controls subcellular localization and possesses several potential phosphorylation sites unique to either RAL-A or RAL-B.

**Fig. 7:**
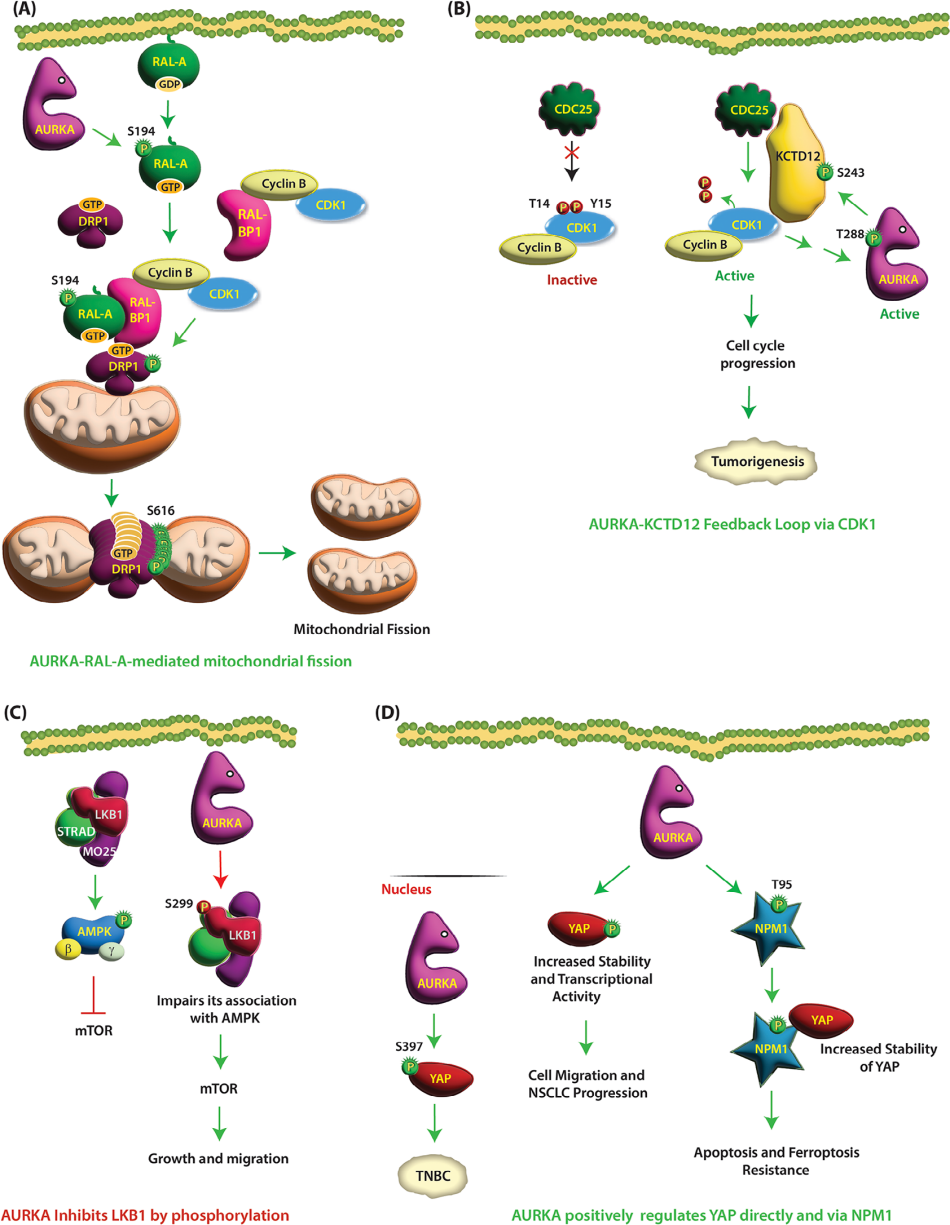
(**A**) AURKA phosphorylates RAL-A at S194, which enhances its activity causing cell migration, anchorage-independent growth and mitochondrial fission. AURKA-mediated RAL-A phosphorylation at S194 causes its relocalization to mitochondrial membranes, where it binds RalBP1 and Drp1. RalBP1 associates with CDK1/cyclin B, which in turn phosphorylates Drp1 at S616 causing its oligomerization and subsequent mitochondrial fission. (**B**) AURKA and KCTD12 are involved in a positive feedback loop. AURKA phosphorylates KCTD12 at S243, which allows its interaction with CDK1. The CDK1-CDC25B-KCTD12 complex enables CDC25-mediated dephosphorylation of CDK1, which activates CDK1. In turn CDK1 activates AURKA via an indirect mechanism. Active AURKA further phosphorylates KCTD12 at S243 to initiate a positive feedback loop that facilitates tumorigenesis. (**C**) AURKA inhibits LKB1’s tumor-suppressive roles via phosphorylation. AURKA physiologically interacts with LKB1 and phosphorylates it at S299, which impairs its association with AMPK, thereby facilitating NSCLC growth and migration. (**D**) AURKA positively regulates YAP by direct phosphorylation and indirectly via NPM1. AURKA and YAP specifically associate in the nucleus, where AURKA phosphorylates it at S397, which increases its transcriptional activity in TNBC. In lung cancer, AURKA increases YAP1 protein levels and transcriptional activity using its kinase activity, although the phosphorylation site was not identified. AURKA directly phosphorylates NPM1 at T95. Phosphorylated NPM1 binds YAP1, which stabilizes the latter, leading to tumorigenesis in ES

AURKA uniquely phosphorylates and activates RAL-A but not RAL-B. Wu et al. initially identified that AURKA directly phosphorylates RAL-A at S194, which enhances its activity by increasing the concentration of GTP-loaded RAL-A (Fig. 7A) (Table 1) [111]. They further showed that ectopic expression of V23Ral-A in AURKA-overexpressing MDCK cells promotes collagen I-induced cell migration and anchorage-independent growth due to S194 phosphorylation. Lim et al. subsequently uncovered that AURKA-mediated RAL-A phosphorylation at S194 activates and translocates it from the plasma membrane to internal membranes, which in turn activate its main effector proteins RalBP1 and Drp1 [112]. As RalBP1 associates with CDK1/cyclin B, it results in Drp1 phosphorylation by CDK1 at S616 causing its oligomerization and subsequent mitochondrial fission (Fig. 7A).

Kashatus et al. further uncovered a direct link between AURKA-mediated Ral-A phosphorylation and mitochondrial fission via RalBP1 and Drp1. Drp1 is a large GTPase, which mediates mitochondrial fission using GTP hydrolysis. However, under normal circumstances, Drp1 is mainly cytoplasmic with only ~ 3% located on the surface of mitochondrial outer membrane. AURKA-mediated RAL-A phosphorylation at S194 triggers its relocalization to mitochondrial membranes, where it recruits both RalBP1 and Drp1 increasing their local concentration. Furthermore, RalBP1 binds Cyclin B, which brings CDK1/cyclin B complex to the close vicinity of Drp1. In turn, CDK1 phosphorylates Drp1 at S616 causing its oligomerization and subsequent mitochondrial fission (Fig. 7A) (Table 1). As both RAL-A and AURKA are overexpressed in cancer, regulation of mitochondrial fission could be one of the mechanisms by which RAL-A fuels cancer [113]. As S194 is not present in RAL-B, this mechanism of mitochondrial translocation is unique to RAL-A.

### **Potassium channel tetramerization domain containing 12** (**KCTD12)**

Potassium channels are transmembrane proteins that can promote cancer by regulating numerous pathological processes including proliferation, migration, invasion, cancer stem cell formation, and angiogenesis via potassium flow across cell membranes. KCTD12 is a component of GABA-B receptors and contributes to presynaptic excitation by GABA-B receptors. KCTD12 overexpression occurs in higher tumor grades in gastrointestinal stromal tumors [114], but it acts as a tumor suppressor in colon cancer [115]. Thus, the role of KCTD12 in cancer remains incompletely understood.

KCTD12 is both a mitotic and cancer target of AURKA. Zhong et al. demonstrated that KCTD12 levels are significantly upregulated in cervical and lung cancers and are associated with larger tumor volumes, advanced pathological stages, and poor survival [116]. Mechanistically, AURKA phosphorylates KCTD12 at S200 and S243, which allows it to form a complex with CDK1 and CDC25B (Fig. 7B) (Table 1). Interestingly, AURKA-mediated phosphorylation of KCTD12 at S243 is essential for its interaction with CDK1, but not with CDC25. The role of S200 phosphorylation remains unclear. The CDK1-CDC25B-KCTD12 complex enables CDC25-mediated dephosphorylation of CDK1 at T14 and Y15, which activates CDK1. In turn CDK1 activates AURKA via an indirect mechanism, which leads to autophosphorylation of AURKA at T288 (Table 2). Active AURKA in turn, phosphorylates KCTD12 at S243 to initiate a positive feedback loop that facilitates mitotic entry and tumorigenesis (Fig. 7B) [116].

### Liver kinase B1 (LKB1)

Liver kinase B1 (LKB1), a serine/threonine kinase, acts as a tumor suppressor. LKB1 is the main kinase that directly phosphorylates AMPK, along with 12 related kinase family members that have critical roles in cell growth, polarity, and metabolism. Activation of LKB1-AMPK signaling with exercise and calorie restriction inhibits the mTOR pathway, which in turn suppresses cancer risk (Fig. 7C). Not surprisingly, it is either lost or has loss of function mutations in non-small cell lung cancers (NSCLC), which is causal for its initiation and progression. AURKA is highly expressed in NSCLC and is associated with poor outcomes in patients [117]. AURKA physiologically interacts with LKB1 and phosphorylates it at S299, which impairs the association of LKB1 with its main substrate, AMPK, which in turn inhibits LKB1’s tumor-suppressive roles, thereby facilitating NSCLC growth and migration (Fig. 7C) (Table 1).

### **Yes-associated protein** (**YAP)**

The Hippo pathway is an evolutionary conserved signaling cascade that functions as a key regulator of cell proliferation, differentiation, and organ size by acting as an integral mechanosensor module. The Hippo pathway has two main modules: the core serine-threonine kinase module, which mainly consists of MST1/2 and LATS1/2; and the transcriptional module, which comprises of transcriptional co-activators including YAP (aka YAP1) and TAZ (transcriptional co-activators with PDZ-binding motif). YAP shuttles between the cytoplasm and nucleus in a signal-dependent manner.

MST1/2 and its binding partner SAV1 (Salvador) phosphorylate and activate LATS1/2 and its binding partner MOB1/2. LATS1/2 in turn phosphorylates YAP at S127, which results in its binding to the 14-3-3 adaptor protein, cytoplasmic retention, and transcriptional inactivation. Similarly, CK1-mediated phosphorylation of YAP1 primes it for polyubiquitylation and degradation [118]. In contrast, nuclear YAP binds DNA-binding transcription factors TEAD to promote the expression of several growth-related genes. Thus, increased expression and/or nuclear accumulation of YAP drives tumor initiation, progression, metastasis, drug resistance, immune evasion, and stemness, underscoring YAP as a potent oncogene and therapeutic target in cancer.

AURKA interacts with YAP in a Hippo-independent manner. Chang et al. showed that aberrant expression of AURKA and YAP promotes triple-negative breast cancer pathogenesis [119]. AURKA and YAP specifically associate in the nucleus, where AURKA phosphorylates it at S397, which increases its transcriptional activity, leading to aggressive oncogenic phenotypes. Interestingly, as previous reports have shown that S397 phosphorylation of cytoplasmic YAP leads to its degradation, the authors concluded that AURKA-mediated phosphorylation of YAP at S397 specifically occurs in the nucleus with opposite outcomes (Fig. 7D) (Table 1).

AURKA-YAP signaling has also been explored in lung cancer [120]. AURKA levels positively correlate with YAP levels in lung cancer. These authors further showed that although AURKA does not regulate the nucleo/cytoplasmic spread of YAP, it increases its protein levels and transcriptional activity in lung cancer cells using its kinase activity. AURKA increases YAP stability by blocking autophagy (Fig. 7D). Surprisingly, unlike Chang et al. study, which showed that the S397 site is an AURKA site, Wang et al. showed that the S397A phospho-dead mutant of YAP does not block the effect of AURKA on YAP, although the exact phosphorylation site was not identified [120]. Nevertheless, YAP is a key downstream effector of AURKA and promotes proliferation and migration in lung cancer cells.

### Nucleophosmin1 (NPM1)

Ewing’s sarcoma (ES) is a highly aggressive round-cell tumor of bone or soft tissue that mainly affects children, adolescents, and young adults. A recent study revealed that AURKA is upregulated in ES and associated with poor prognosis [121]. AURKA promotes tumorigenesis in vivo, and its inhibition induces apoptosis and ferroptosis in ES cells. Mechanistically, AURKA regulates ES cell proliferation and resistance to apoptosis and ferroptosis by directly phosphorylating NPM1 at T95 (Table 1) (Fig. 7D). NPM1 is a multifunctional nucleolar protein, which is involved in many cellular processes including ribosome biogenesis, rRNA transcription and histone chaperone activity. Interestingly, T-95 phosphorylated NPM1 directly interacts with YAP1, which stabilizes the latter, leading to tumorigenesis in ES (Fig. 7D).

As noted above, Wang et al. observed that that the AURKA kinase activity is required for stabilizing YAP1 in lung cancer but did not identify the phosphorylation site [120]. Therefore, it is possible that like ES, in lung cancer, AURKA may stabilize YAP1 indirectly by phosphorylating NPM1. Future studies are needed to fully uncover these mechanisms (Fig. 7D).

### Ribosomal protein S6 kinase B1 (RPS6KB1)

RPS6KB1 (aka p70S6K) is a mitogen-activated serine/threonine protein kinase that controls diverse cellular processes, including cell growth, survival, mRNA transcription and processing, glucose homeostasis, and protein synthesis. RPS6KB1 is constitutively activated in many human malignancies, underscoring its potential as a therapeutic target. Its phosphorylation at T389 is essential for its catalytic activity. Accordingly, in NSCLC, hyperphosphorylation of RPS6KB1 at T389, but not its expression level, correlates with poor prognosis [122].

**Fig. 8:**
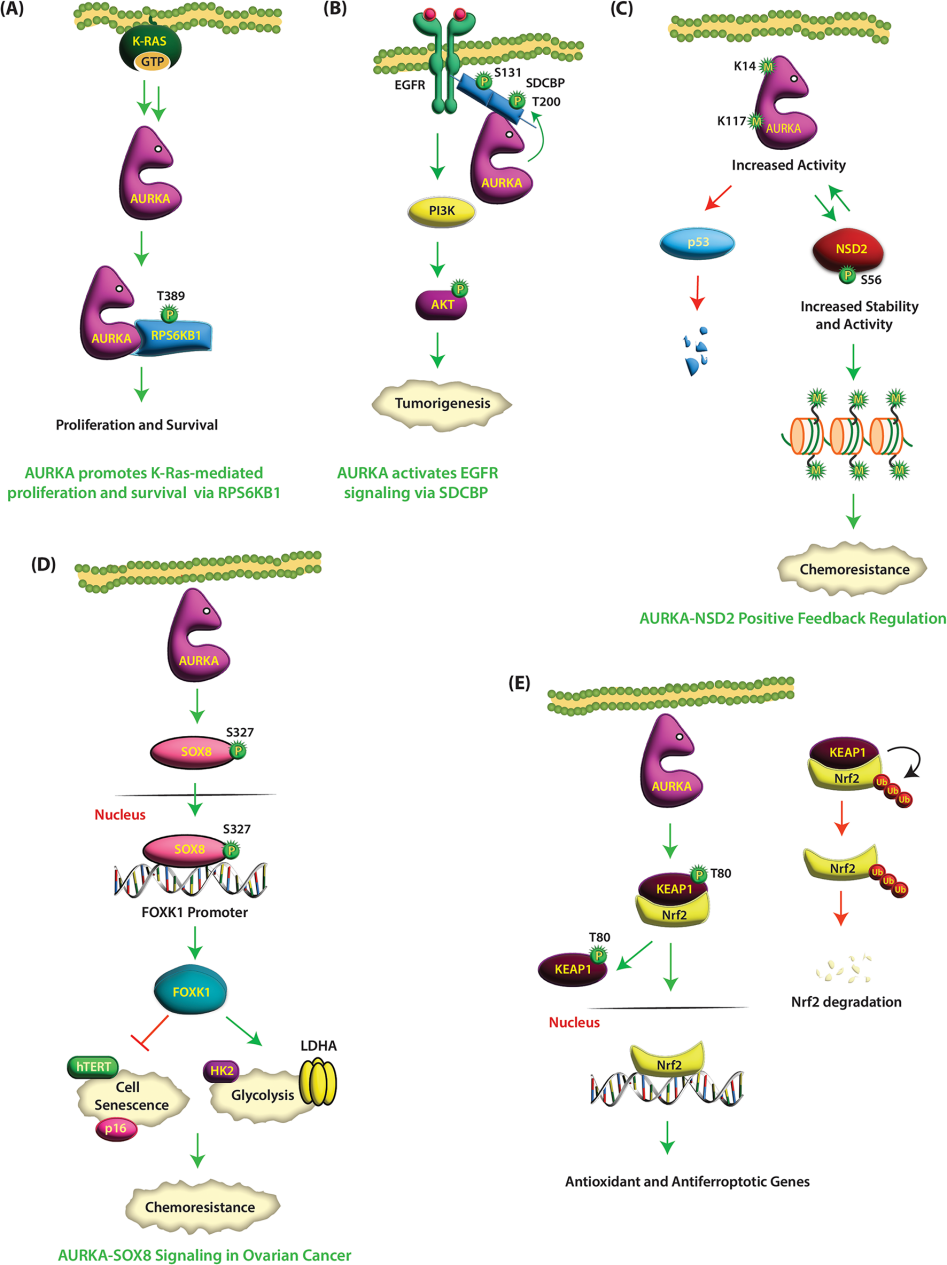
(**A**) AURKA phosphorylates RPS6KB1 is mutant K-Ras expressing cells. AURKA colocalizes and directly phosphorylates RPS6KB1 at T389 in K-Ras mutant gastrointestinal cancer cells leading to increased proliferation and survival. (**B**) AURKA activates EGFR signaling via SDCBP. AURKA stabilizes SDCBP by phosphorylating it at the S131 and T200 sites. Furthermore, phosphorylated SDCBP binds EGFR and prevents its internalization. Sustained EGFR signaling constitutively activates the PI3K-AKT pathway to promote highly oncogenic signaling in ESCC. (**C**) NSD2 and AURKA positively regulate each other. NSD2 interacts with the N-terminal of AURKA and methylates it at K14 and K117. AURKA methylation increases its kinase activity, and induces its binding to p53, which triggers p53 degradation. AURKA also phosphorylates NSD2 at S56, which prevents its degradation and increases its activity in a feedback loop. (**D**) AURKA promotes chemoresistance via SOX8. AURKA phosphorylates SOX8 at S327, which increases its nuclear localization. Nuclear SOX8 increases FOXK1 transcription by binding its promoter. FOXK1 promotes chemoresistance, suppresses cell senescence, and promotes glucose metabolism by regulating specific genes. (**E**) AURKA promotes ferroptosis resistance via KEAP1 phosphorylation. AURKA binds and phosphorylates KEAP1 at T80, which results in Nrf2 dissociation, nuclear translocation and activation of anti-ferroptosis and antioxidant genes. In the absence of AURKA-mediated phosphorylation, KEAP1 binding to Nrf2 leads to its degradation

Wang-Bishop et al. revealed RPS6KB1 as a direct target of AURKA in mutant K-Ras activated gastrointestinal cancer cells [123]. KRAS is mutated at codon 12 in most cancers, including pancreatic, colorectal, gastric, and esophageal adenocarcinomas. Mutant K-Ras is constitutively active and is associated with drug resistance, metastasis, and poor clinical outcomes. AURKA colocalizes and directly phosphorylates RPS6KB1 at T389 in K-Ras mutant gastrointestinal cancer cells, but not in WT-K-Ras expressing cells, which promotes tumorigenesis (Table 1). Thus, AURKA is a key downstream effector of mutant K-Ras that enhances oncogenicity via RPS6KB1 (Fig. 8A).

### SDCBP

Syndecan Binding Protein (SDCBP) is an adapter protein possessing two tandem PDZ domains, which promote tumorigenesis and metastasis in many human cancers. SDCBP is frequently overexpressed in esophageal squamous cell carcinoma (ESCC) tissues as compared to controls, is strongly correlated with late clinical stage, and predicts poor prognosis in patients [124]. AURKA not only stabilizes SDCBP by phosphorylating it at the S131 and T200 sites, but the phosphorylation of these sites also allows SDCBP to bind EGFR and prevent its internalization (Table 1). Sustained EGFR signaling activates the PI3K-AKT pathways to promote highly oncogenic signaling in ESCC (Fig. 8B).

### Multiple myeloma SET domain containing protein (MMSET aka NSD2)

Multiple myeloma (MM) ranks second among all hematological malignancies. Bortezomib, a first-in-class proteasome inhibitor, is an approved drug for MM, either alone or in combination with other drugs. However, MM patients suffer from a poor prognosis due to high intrinsic or acquired drug resistance. AURKA is highly expressed in MM and associated with strong drug resistance. Not surprisingly, AURKA inhibitors show strong synergy with bortezomib in MM cells. AURKA inhibitors are currently in Phase II clinical trials for relapsed or refractory MM patients.

Recently, NSD2 (aka MMSET), a methyl transferase, was identified as a direct target of AURKA in MM, which unraveled the mechanism by which the AURKA-NSD2 feedback loop promotes chemoresistance. NSD2 is robustly expressed in all MM patients, which is a high-risk factor and essential for MM pathogenesis. NSD2 and AURKA are involved in a positive feedback loop, which fuels MM pathogenesis. NSD2 interacts with the N-terminal of AURKA and methylates it at K14 and K117 in vitro and in vivo (Fig. 7C) (Table 2) [125]. AURKA methylation not only increases its kinase activity, but also induces its binding to p53, which leads to the proteasomal degradation of p53, increasing oncogenic phenotypes. Furthermore, NSD2 depletion potently inhibits cancer cells and renders them sensitive to growth inhibition by AURKA inhibitor alisertib [125]. A subsequent study revealed that AURKA directly phosphorylates NSD2 at S56, which prevents its degradation and increases its methyltansferase activity (Table 1) [126]. Thus, AURKA methylation by NSD2 and AURKA-mediated phosphorylation of NSD2 bilaterally form a positive regulating loop leading to chemoresistance in MM (Fig. 8C) [126].

### SRY-box 8 (SOX8)

SOX8 is a transcription factor that belongs to the Sex-Determining Region Y-Box family of proteins. SOX8 facilitates chromatin loosening to increase the transcription of specific genes. SOX8 plays important roles in the regulation of embryonic development, including tissue specification, organ development, stem cell homeostasis and maintenance of male fertility. SOX8 deregulation has been implicated in multiple diseases, including cancer. Sun et al. showed that AURKA and SOX8 are significantly upregulated in cisplatin-resistant and cisplatin-sensitive ovarian cancer tissues and associated with poor survival [127]. AURKA directly binds SOX8 and phosphorylates it at S327, which increases its nuclear localization (Fig. 8D) (Table 1). RNA-Seq experiments further revealed that nuclear SOX8 increases the transcription of FOXK1 by binding its promoter. In turn, FOXK1 promotes chemoresistance, suppresses cell senescence, and promotes glucose metabolism by regulating genes related to cell senescence and glycolysis, including hTERT, P16, LDHA, and HK2. Thus, the AURKA/SOX8/FOXK1 signaling axis promotes chemoresistance by suppressing cell senescence and inducing glucose metabolism in ovarian cancer (Fig. 8D).

### Kelch-like ECH-associated protein 1 (KEAP1)

Keap1 is an adaptor subunit of Cullin-3 E3 ubiquitin ligase, which binds transcription factor Nrf2 (nuclear factor erythroid 2-related factor 2) and promotes its degradation via ubiquitylation. In the absence of Keap1, Nrf2 translocates to the nucleus and mediates antioxidant response element (ARE)-dependent gene expression, thereby maintaining the cellular homeostasis. Under physiological and premalignant conditions, Nrf2 activation is cancer preventive, but in malignant situations, Nrf2 activation promotes chemoresistance and tumorigenesis [128].

AURKA binds and phosphorylates KEAP1 at T80, which results in Nrf2 dissociation, nuclear translocation and activation of anti-ferroptosis genes in human meningioma cell lines [129]. AURKA is highly expressed in high grade meningoma, which is one of the most prevalent primary central nervous system tumors. AURKA-mediated KEAP1 phosphorylation enhances tumorigenesis and ferroptosis resistance via Nrf2 stabilization. Thus, combination therapy using alisertib and erastin (a ferroptosis-inducing agent) was significantly effective in the murine model of meningioma [129].

## Discussion

AURKA is predominantly recognized as a mitotic kinase. Not surprisingly, numerous mitotic targets of AURKA have uncovered the signaling cascades by which it plays an indispensable role during mitosis. However, recent studies have also uncovered critical contributions of interphasic AURKA in DNA replication, regulation of cell cycle progression, and maintenance of genomic stability [130]. Additionally, it has been implicated in processes such as centrosome maturation, microtubule organization, and cell migration, highlighting its multifaceted functions beyond mitosis.

While AURKA amplification is the critical initiating upstream event in causing cancer, increased transcription or stabilization of AURKA protein in many cancers can also lead to its upregulation and activation. The mechanisms behind AURKA protein upregulation in the absence of amplification have not been extensively studied. Therefore, in these cases AURKA upregulation is still considered as the primary event causing oncogenesis. AURKA is overexpressed in a cell cycle-independent manner and is mislocalized in the cytoplasm and/or nucleus, which allows it to phosphorylate numerous pathological substrates promoting oncogenesis. This review specifically focuses on 33 of such direct cancer-specific targets of AURKA and the signaling cascades by which it promotes oncogenic phenotypes.

One of the common themes that emerges from the analysis of these 33 direct cancer targets, is that AURKA is often engaged in a feedback loop with its substrates, which could be the decisive factor causing its sustained upregulation and hyperactivation in cancer cells, an Achilles heel not exploited before. AURKA engages in a negative feedback loop with many tumor suppressors including PHLDA1, SPOP, NKX3.1, VHL, p53 and BRCA1, which in turn reciprocate by negatively regulating AURKA activity and/or levels in a feedback loop (Table 2). Accordingly, VHL loss in the vast majority of ccRCC may cause AURKA upregulation by increasing its transcription via β-catenin and by inhibiting its degradation. Not surprisingly, VHL loss in ccRCC cells renders them highly sensitive to AURKA inhibitors. Similarly, p53, SPOP, NKX3.1, FAF1 and BRCA1 loss are common in cancers of various origins, which may initiate or potentiate AURKA deregulation. Importantly, FAF1 phosphorylation by AURKA does not regulate FAF1 levels, but phosphorylated FAF1 triggers AURKA ubiquitylation, indicating that FAF1 loss may cause AURKA upregulation in tumor tissues.

Likewise, AURKA, and its oncogenic substrates are often independently reported to be concomitantly increased in many cancers. AURKA is involved in a positive feedback loop with many oncogenes including LIMK2, TWIST1, YBX1, NSD2, ALDH1A and KCTD12 (Table 2). LIMK2, TWIST1, YBX1 and ALDH1A1 stabilize AURKA protein levels in reciprocation. NSD2 activates AURKA kinase activity by methylation. However, this methylation event also allows AURKA to bind p53 and degrade it. As p53 inhibits AURKA transcription and degrades AURKA protein via FBXW7 (Fig. 2A), p53 degradation due to NSD2-mediated AURKA methylation indirectly causes AURKA overexpression as well. As NSD2 is an essential and high-risk factor for MM pathogenesis, it may also serve as an upstream activator and stabilizer for AURKA in MM. We have shown that LIMK2 levels increase with prostate cancer progression, with the highest levels in CRPC [96]. Similarly, ALDH1A1 and TWIST1 are highly upregulated in cancer stem cells. Thus, upregulation of such oncogenic targets could be the primary event causing AURKA upregulation in these cancers, which in turn engages in a feedback loop with its targets. We propose that the dynamic interplay between AURKA and its substrates offers innovative opportunities for targeted therapeutic interventions. By targeting these substrates, it may be possible to disrupt this feedback loop to effectively reverse AURKA levels, thereby providing a promising avenue for developing safer AURKA-targeted therapeutics with minimal collateral toxicity.

Numerous mitotic targets of AURKA have been uncovered to date. A few of these mitotic targets could also serve as cancer targets either due to their overexpression and/or mislocalization along with AURKA in specific cancers. An example includes KCTD12, which is a mitotic target but upon overexpression in cervical and lung cancers promotes tumorigenesis in an AURKA-dependent manner. Interestingly, AURKA-mediated phosphorylation of KCTD12 does not affect KCTD12 levels or activity, instead AURKA uses KCTD12 to activate itself via CDK1. Similarly, NuMa is overexpressed in many tumors and is strongly correlated with poor prognosis. AURKA-mediated phosphorylation of NuMa inhibits apoptosis and promotes proliferation, suggesting that it might also serve as a cancer target of AURKA.

Nearly fifty AURKA inhibitors are in clinical trials, but no AURKA-targeted drug has been approved yet, partly due to associated collateral toxicity including neutropenia, somnolence and mucisitis. Conversely, only about ten AURKB inhibitors are in clinical trials, and most of them are still in Phase I clinical trial. Alisertib, one of the most selective and extensively studied AURKA inhibitors has entered Phase III clinical trial, although it has shown limited efficacy in a wide range of tumors as a single agent. Alisertib was modest as a single agent in Phase 2 clinical trials involving peritoneal carcinoma, acute myelogenous leukemia, fallopian tube cancer, ovarian cancer, and high-grade myelodysplastic syndrome [131]. In another phase II trial, alisertib exhibited 18% response in breast cancer patients, 21% in small-cell lung cancer patients, 4% in patients with non-small-cell lung cancer, and 9% in both head and neck squamous-cell carcinoma, and gastro-esophageal adenocarcinoma. Furthermore, serious adverse events including neutropenia, anemia and leukopenia were reported in 43% of patients [132].

These findings suggest that AURKA inhibitors could be more effective in combination with other targeted therapies, leading to improved tumor response rates. Indeed, AURKA inhibition shows significantly improved overall survival and progression-free survival in combination with chemotherapeutic drugs in clinical trials [133]. Similarly, AURKA inhibition is synthetically lethal in tumors lacking certain tumor suppressors, which can be utilized to develop highly effective cancer-specific drugs. Accordingly, AURKA inhibition in RB1-deficient lung cancer is synthetically lethal [134]. Likewise, AT-rich interactive domain 1 A (ARID1A) is a tumor suppressor, which is frequently inactivated in many cancers. AURKA inhibition or depletion is synthetically lethal in ARID1A-deficient colorectal cancer (CRC) cells as well [135]. Further research on identifying biomarkers that can predict response to AURKA inhibitors can help in selecting patients who are more likely to benefit from this treatment approach.

Importantly, despite numerous known cancer-specific substrates of AURKA, it has not been targeted in combination with its substrates. This multimodal approach has the potential to be highly synergistic due to the reciprocal feedback loop between AURKA and its substrates. In addition to developing dual inhibitors that simultaneously block the activity of both AURKA and its cancer-driving substrates, another approach could involve designing molecules that specifically disrupt the interaction between AURKA and its substrates, thereby inhibiting their oncogenic functions. Indeed, alisertib binding to AURKA induces a conformational change in its activation loop that disrupts its binding to N-Myc, causing N-Myc degradation and tumor regression [136]. Although N-Myc is not a phosphorylation target of AURKA, AURKA-N-Myc binding is highly synergistic causing extreme oncogenicity. Thus, in a subset of castration-resistant neuroendocrine prostate cancer patients, which had both AURKA and N-Myc overactivity responded exceptionally well when treated with alisertib [137].

Additionally, combination therapies involving AURKA inhibitors and agents that could upregulate specific tumor suppressor substrates should also be explored. In this regard, several small molecule inhibitors have entered clinical trials that target p53-MDM2 interactions, which stabilize p53, inducing cancer cell death (Table 3) [138]. Furthermore, as p53 is often mutated in cancer, several allele-specific mutant p53 rescue compounds have entered clinical trials, which restore mutant p53 function. Interestingly, R175G mutation in p53 is oncogenic, prompting the development of an RNA aptamer-based proteolysis targeting chimera (PROTAC) for its selective degradation (Table 3) [139]. As p53 and AURKA are involved in a negative feedback loop, it would be interesting to investigate the combination of restoring p53 activity with AURKA inhibition. Alternatively, AURKA inhibitors are expected to work synergistically with R175G-p53 PROTAC.

**Table 3:** Targeting of downstream substrates of AURKA

	Druggable AURKA Substrates			
	Substrate	Targeting	Role	Reference
1	p53	Small molecule inhibitor	Several inhibitors are in clinical trial but none is yet approved by FDA	[138], [139]
		PROTAC	RNA aptamer-based PROTAC was developed that selectively targets p53‐R175H, most common p53 hotspot mutation	
2	hnRNPK	Small molecule inhibitor	small molecule inhibitor (hnRNPK-IN-1) disrupts the binding of hnRNPK and c-myc promoter and inhibits c-myc transcription.	[140]
3	VHL	Small molecule inhibitor PROTAC	Several VHL inhibitors and VHL-based PROTACs are available	[141]
4	FAF1	Small molecule inhibitor	KM-819 is a novel FAF1 inhibitor under clinical development for the treatment of Parkinson’s disease	[142]
5	ERα	Small molecule inhibitor	Several FDA approved drugs are available	[143], [144]
		PROTAC	ERD-148 is a potent and selective PROTAC ERα degrader.	
6	GSK3β	Small molecule inhibitor	Many GSK-3β inhibitors have been synthesized, but only few have entered clinical trials.	[145], [146]
		PROTAC	Several set of GSK-3β PROTAC are currently available	
7	β-Catenin	Small molecule inhibitor	Many small-molecule inhibitors present but none of them approved for the clinical use	[147], [148]
		PROTAC	xStAx-VHLL is a potent PROTAC β-catenin degrader	
8	HDAC6	Small molecule inhibitor	Several HDAC6 inhibitors are in clinical trials but none approved by the FDA for cancer therapy.	[149], [150]
		PROTAC	Several HDAC6-targeting PROTACs are present	
9	ALDH1A1	Small molecule inhibitor	Several molecular inhibitors are present but none of them has passed clinical trial	[151]
10	YBX1	Small molecule inhibitor	SU056 is the only small molecule inhibitor of YB-1 which inhibits the proliferation of ovarian cancer cells.	[152]
11	LIMK2	Small molecule inhibitor	Several small molecule inhibitors are in preclinical stage but only one of them (LX7101) has reached clinical trial.	[153]
12	SPOP	Small molecule inhibitor	Several SPOP molecular inhibitors are in preclinical stage	[154]
13	SOX2	PROTAC	a series of SOX2-targeting bioPROTACs, are being developed by EPD Biotherapeutics for degradation of SOX2 protein	[155]
14	LDHB	Small molecule inhibitor	Several LDHB inhibitor are being tested in preclinical stage.	[156], [157]
		PROTAC	MS6105 is the first LDH PROTAC that degrades both LDHA and LDHB in a time- and ubiquitin-proteasome system-dependent manner	
15	RAL-A	Small molecule inhibitor	A number of small molecule inhibitors exist that can bind to an allosteric site found in the inactive GDP-bound states of RALA and RALB.	[158]
16	YAP	Small molecule inhibitor	Multiple approaches and inhibitors have been formulated to target YAP/TAZ, and their interaction with TEAD transcription factors	[159], [160]
		PROTAC	YZ-6 is a YAP PROTAC which promotes ubiquitination and subsequent degradation of YAP	
17	NPM1	Small molecule inhibitor	Several NPM1 inhibitors are under different phases of clinical development	[161]
18	RPS6KB1	Small molecule inhibitor	Rapamycin (sirolimus) and rapalogs (everolimus, temsirolimus) has been approved by FDA while several other inhibitors are under different phases of clinical development.	[162]
19	SDCBP	Small molecule inhibitor	Small molecule inhibitor of SDCBP work by disrupting the interaction between SDCBP and its binding partners	[163]
20	MMSET aka NSD2	Small molecules inhibitor	Several small molecule inhibitors of NSD are undergoing preclinical and clinical investigation	[164]
		PROTAC	PROTAC strategies have been explored for the targeted degradation of NSD proteins	
21	KEAP1	Small molecules inhibitor PROTAC	Several KEAP1 inhibitor and KEAP1 PROTAC are undergoing preclinical study	[165]
**AURKA Substrates that lack targeting agents**				
	**Substrate**	**Targeting**		
22	BRCA1	no small molecule inhibitor or PROTAC is currently available		
23	PHLDA1	no small molecule inhibitor or PROTAC is currently available		
24	RASSF1A	no small molecule inhibitor or PROTAC is currently available		
25	IκBα	no small molecule inhibitor or PROTAC is currently available		
26	TIFA	no small molecule inhibitor or PROTAC is currently available		
27	HURP	no small molecule inhibitor or PROTAC is currently available		
28	TWIST1	no small molecule inhibitor or PROTAC is currently available		
29	NKX3.1	no small molecule inhibitor or PROTAC is currently available		
30	NuMa	no small molecule inhibitor or PROTAC is currently available		
31	KCTD12	no small molecule inhibitor or PROTAC is currently available		
32	LKB1	no small molecule inhibitor or PROTAC is currently available		
33	SOX8	no small molecule inhibitor or PROTAC is currently available		

Heterogeneous nuclear ribonucleoprotein K (hnRNPK); Pleckstrin homology-like domain, family A, member 1 (PHLDA1); Ras-associated domain family 1 isoform A (RASSF1A); Von Hippel Lindau (VHL); Inhibitors of NF-κB (IκBs); TRAF-interacting protein with the FHA domain (TIFA); FAS-associated Factor-1 (FAF1); Hepatoma upregulated protein (HURP); Glycogen synthase kinase (GSK)3β; Aldehyde dehydrogenase 1 (ALDH1A1); Y-box binding protein-1 (YBX1); LIM-domain kinase-2 (LIMK2); Nuclear mitotic apparatus (NuMa); Lactate dehydrogenase B (LDHB); Ral small GTPase (RalA); C-terminus of HSP70-interacting protein (CHIP); Potassium channel tetramerization domain containing 12 (KCTD12); Liver kinase B1 (LKB1); Yes-associated protein (YAP); Syndecan binding protein (SDCBP); Ribosomal protein S6 kinase B1 (RPS6KB1); Nucleophosmin1 (NPM1); Multiple Myeloma SET Domain Containing Protein (MMSET); Sex-determining region Y (SRY)-Box 8 (SOX8); Kelch-like ECH-associated protein 1 (KEAP1)

A small molecule inhibitor for FAF1 is in clinical development for Parkinson’s Disease, however, no FAF1 stabilizer or activator is known to date (Table 3). As FAF1 negatively regulates AURKA, FAF1 stabilizer or activator is expected to work synergistically with AURKA inhibitors. Furthermore, many small molecule inhibitors have been developed against several AURKA substrates that function as oncogenes, including LIMK2, ERα, GSK3β, β-catenin, ALDH1A1, YBX1, RAL-A, YAP, NPM1, NSD2 and RPS6KB1 (Table 3). Future studies are needed to identify potential synergistic effects between AURKA inhibitors in combination with one or more of its substrates’ inhibitors/activators in distinct genetic backgrounds. This would allow for the rational design of combination therapies tailored to individual cancer profiles. Additionally, leveraging genomic and proteomic profiling could help identify specific biomarkers that predict responsiveness to these combinations, enabling a more personalized and targeted cancer therapy strategy. Developing such effective combination therapies may thus unlock the full potential of AURKA as a critical drug target in cancer treatment.

## Data Availability

No datasets were generated or analysed during the current study.

## Abbreviations

AKT: RAC-alpha serine/threonine-protein kinase
ARID1A: AT-rich interactive domain 1 A
AURKA: Aurora Kinase A
APC: Anaphase‑promoting complex
AR: Androgen receptor
ALDH1A1: Aldehyde dehydrogenase-1A1
BRCA: Breast cancer susceptibility protein
ccRCC: clear cell renal cell carcinoma
CDC25: Cell division cycle 25
CDK1: Cyclin-dependent kinase-1
Chfr: Checkpoint With Forkhead And Ring Finger Domains
CEP192: Centrosomal protein-192
CSC: Cancer stem phenotype
Drp1: Dynamin related protein 1
EMT: Epithelial Mesenchymal Transition
ERα: Estrogen receptorα
ERK: Extracellular signal-regulated kinase
ESCC: Esophageal squamous cell carcinoma
FAF1: FAS-associated Factor-1
FBXW7: F-box and WD repeat-containing 7
GSK3β: Glycogen Synthase Kinase clear cell 3β
HDAC1: histone deacetylase 1
HDAC6: Histone deacetylase 6
hnRNPK: Heterogeneous nuclear ribonucleoprotein K
HURP: Hepatoma upregulated protein
IDH1: Inhibitor of differentiation 1
IκBα: Nuclear factor of kappa light polypeptide gene enhancer in B-cells inhibitor alpha
KEAP1: Kelch-like ECH-associated protein 1
KCTD12: Potassium Channel Tetramerization Domain Containing 12
LDHB: Lactate dehydrogenase B
LIMK2: LIM Kinase 2
LKB1: Liver kinase B1
MM: Multiple myeloma
MMSET aka NSD2: Multiple Myeloma SET Domain Containing Protein
MT: Microtubules
NKX3.1: NK3 homeobox 1
NEDD9 aka HEF1: Neural precursor cell expressed developmentally downregulated 9
NF: κB-nuclear factor-kappa B
NPM1: Nucleophosmin1
Nrf2: Nuclear factor erythroid 2-related factor 2
NSCLC: Non-small cell lung cancer
NuMa: Nuclear mitotic apparatus
PHLDA1: Pleckstrin homology-like domain, family A, member 1
PLD2: Phospholipase D2
PP6C: Protein phosphatase 6 C
PKA: Protein kinase A
PROTAC: Proteolysis targeting chimera
RASSF1A: Ras-associated domain family 1 isoform A
RAL: A-RAS like proto-oncogene A
RalBP1: RalA binding protein 1
Rb1: Retinoblastoma 1
RPS6KB1: Ribosomal protein S6 kinase B1
SAV1: Salvador
SDCBP: Syndecan Binding Protein
SOX2: SRY-box transcription factor 2
SOX8: SRY-box transcription factor 8
SPOP: Speckle-type POZ (pox virus and zinc finger) protein
TAZ: Transcriptional co-activators with PDZ-binding motif
TCHP: Trichoplein
TEAD: TEA domain transcription factor
TIFA: TRAF-interacting protein with the FHA domain
TNBC: Triple-negative breast cancer
TPX2: Targeting protein for Xklp2
VHL: Von Hippel Lindau
YAP: Yes-associated protein
YBX1: Y-box binding protein 1
